# Traditional Chinese medicine constitution and cardiometabolic multimorbidity: a nationwide cross-sectional study in older adults

**DOI:** 10.3389/fpubh.2026.1723708

**Published:** 2026-01-26

**Authors:** Ran Chen, Hou-Qin Li, Jing Xia, Fei-Yu He, Yan Zhang, Zheng Wang, Hong-Yi Zhang, Yao-Ming Yang, Ming-Hua Bai, Cheng Ni

**Affiliations:** 1 School of Chinese Medicine/Center for Studies in Constitution and Reproductive Sciences of Traditional Chinese Medicine, Beijing University of Chinese Medicine, Beijing, China; 2 Department of Geriatrics, Yangzhou Traditional Chinese Medicine Hospital, Yangzhou, China; 3 Dongzhimen Hospital, Beijing University of Chinese Medicine, Beijing, China; 4 Wangqi Academy/National Institute of Traditional Chinese Medicine Constitution and Preventive Medicine, Beijing University of Chinese Medicine, Beijing, China

**Keywords:** cardiometabolic multimorbidity, nationwide cross-sectional study, older adults, personalized prevention, risk stratification, traditional Chinese medicine constitution

## Abstract

**Objective:**

Cardiometabolic multimorbidity (CMM), defined as the coexistence of two or more cardiometabolic diseases, is increasingly prevalent in older adults. Traditional Chinese medicine (TCM) constitution may influence individual susceptibility and provide complementary approaches for prevention and management. This study aimed to examine the potential association between TCM constitution and CMM to offer novel insights into individualized risk stratification and potential preventive approaches for older adults.

**Methods:**

A national cross-sectional study of 24,812 Chinese adults aged ≥60 years was conducted. CMM was defined as having at least two cardiometabolic conditions, including diabetes, stroke, and heart disease. TCM constitutions were assessed using the Chinese Medicine Constitution Questionnaire for the Elderly Edition (CCMQ-EE) and categorized as balanced or eight unbalanced types. The association between TCM constitutions and CMM was analyzed using multivariate logistic regression. To assess the robustness of these associations, inverse probability of treatment weighting (IPTW) based on propensity scores was applied. Stratified analyses assessed subgroup heterogeneity, while tetrachoric correlation and association rule analyses identified constitution co-occurrence patterns.

**Results:**

Qi-deficiency constitution (QDC) [OR 1.57, 95% CI 1.21–2.04], Yang-deficiency constitution (YaDC) [OR 1.63, 95% CI 1.35–1.96], Yin-deficiency constitution (YiDC) [OR 1.62, 95% CI 1.33–1.96], and Phlegm-dampness constitution (PDC) [OR 1.40, 95% CI 1.17–1.68] were independently associated with CMM. Associations remained robust after IPTW based on propensity scores. Subgroup analyses showed a stronger association between YaDC and CMM among obese individuals, while the association between YiDC and CMM was more pronounced in participants with central obesity and without hypertension (P for interaction = 0.049). Common mixed constitution patterns in CMM included YiDC with Dampness-heat constitution (DHC), YiDC with Blood stasis constitution (BSC), and PDC with DHC.

**Conclusion:**

QDC, YaDC, YiDC, and PDC were independently associated with CMM, suggesting that they may represent potential risk factors for its development. Incorporating constitution assessment into routine health evaluations could facilitate the early identification of high-risk subgroups and support the implementation of targeted, constitution-based prevention and management strategies, thereby contributing to reducing the prevalence and burden of CMM in older adults.

## 1 Introduction

With the rapid aging of the global population and increasing life expectancy, the coexistence of multiple chronic conditions—referred to as multimorbidity—has emerged as a major global health challenge (1). Among these, cardiometabolic multimorbidity (CMM), defined as the coexistence of two or more cardiometabolic diseases (CMDs), represents one of the most prevalent and clinically concerning patterns (2). CMM typically includes diabetes, stroke, and cardiovascular disease, and its prevalence has been steadily increasing worldwide (3). For example, in a large longitudinal cohort study of 1,038,704 Chinese adults, the prevalence of CMM rose with age, reaching 5.2% among those aged ≥40 years and 11.6% among those aged ≥60 years (4). CMM substantially increases the risk of cardiovascular events, organ dysfunction, and premature death in older adults. Importantly, epidemiological evidence shows that by age 60, having two CMDs reduces life expectancy by approximately 12 years (5), while three CMDs reduce it by 15 years (1). Collectively, the rising burden of CMM not only undermines health and longevity but also places profound medical, social, and economic demands on healthcare systems, highlighting the urgent need for effective preventive and management strategies.

Despite advances in managing individual cardiometabolic conditions, treating older adults with CMM remains highly challenging. Current clinical guidelines are primarily disease-specific and may provide conflicting recommendations when multiple conditions coexist, complicating therapeutic decision-making (6). Polypharmacy is common, increasing the risk of drug–drug interactions, adverse drug reactions, and poor adherence (7, 8). Given these challenges, complementary approaches that account for individual heterogeneity are urgently needed to improve prevention, risk stratification, and management of CMM in older adults.

Traditional Chinese medicine (TCM) identifies patients’ symptoms and constitutions to develop personalized interventions, demonstrating unique advantages in the prevention and treatment of chronic diseases such as diabetes with coronary heart disease (9, 10). TCM constitution, as an essential branch of TCM, reflects individual variations in physiological, psychological, and metabolic characteristics, and has been widely used in China to explain differential susceptibility to diseases and variation in treatment responses (11, 12). Importantly, TCM constitution identification has been incorporated into China’s public health system as a community-level strategy for personalized prevention, treatment, and management of chronic diseases (13). Growing evidence further suggests that specific constitution types are linked to cardiometabolic risk factors, including hypertension, dyslipidemia, and insulin resistance (14–16). However, most existing studies have concentrated on associations between TCM constitutions and single cardiometabolic diseases, such as diabetes, coronary heart disease, or stroke (17–19), leaving the relationship between constitution types and CMM in older adults poorly understood. Clarifying these associations may enable early identification of vulnerable subgroups, facilitate targeted prevention and intervention strategies, and provide complementary approaches to optimize the management of CMM in older adults.

Given the high prevalence and substantial clinical burden of CMM in aging populations, coupled with the limitations of conventional disease-specific management strategies, there is an urgent need to explore complementary approaches that account for individual heterogeneity. TCM constitution provides a unique framework for such exploration, yet large-scale evidence linking constitution types to CMM is lacking. To address this gap, this study utilized a national cross-sectional dataset of older Chinese adults to investigate the associations between TCM constitution types and CMM. Specifically, we aimed to (i) characterize the distribution of TCM constitution types among older adults with CMM, (ii) evaluate the independent associations between constitution types and CMM using robust statistical approaches, and (iii) explore constitution co-occurrence patterns through correlation and association rule analyses. By integrating TCM constitution theory with modern epidemiological methods, this study seeks to provide novel insights into individualized risk stratification and potential preventive strategies for older adults with CMM.

## 2 Materials and methods

### 2.1 Study design and participants

**Figure 1:**
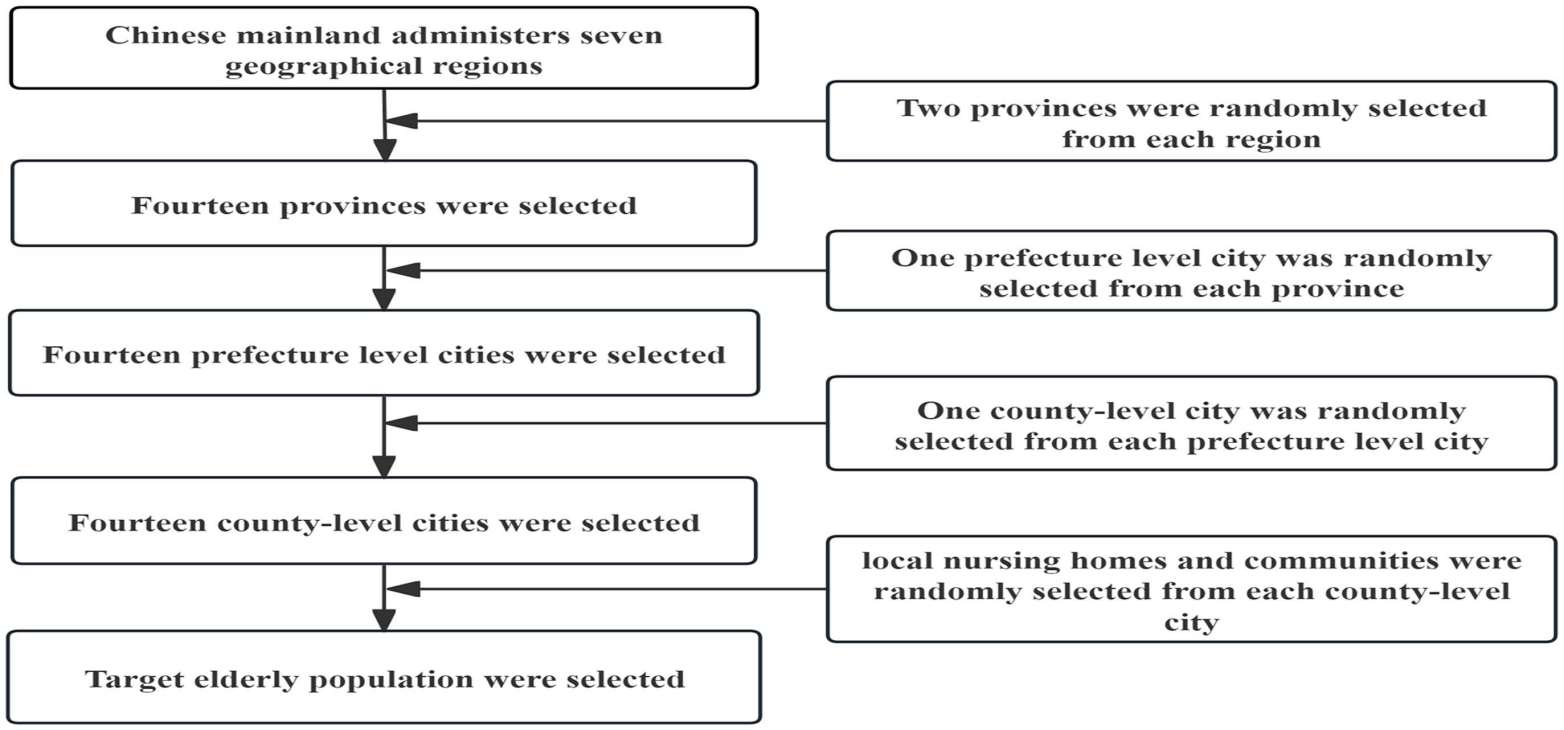
Flowchart of the sampling process.

We used a multi-stage whole-cluster random sampling method to enroll a nationally representative sample of older adults aged 60 years and older from local nursing homes and communities across all seven major regions of mainland China (Northeast, North, Northwest, East, Central, South, and Southwest), spanning the period from January 2020 to November 2023. In the first phase, two provinces were selected randomly from each of China’s seven major geographical areas. In the second phase, one prefecture-level city was selected randomly from each province region. The third phase involved the random selection of one district or county from each selected prefecture-level city. Within these final districts and counties, convenient sampling was utilized based on the capacities of local nursing homes and communities to recruit participants. The overall sampling process is illustrated in Figure 1.

The inclusion criteria were as follows: (i) Chinese nationality, (ii) living in their current residence for 1 year or longer, (iii) age ≥ 60 years (for this study, older adults were defined as individuals aged 60 years or older, according to the World Health Organization and the Chinese government standards for aging population classification), and (iv) signing the informed consent. And exclusion criteria were as follows: individuals who (i) suffered from severe mental illnesses or serious disorders of consciousness, (ii) impaired their ability to understand the survey content, (iii) experienced an acute exacerbation of chronic conditions or those afflicted with acute illnesses, including but not limited to acute myocardial infarction, acute abdominal diseases, and diabetic ketoacidosis, and (iv) the investigation cannot be completed.

This study was approved by the Ethics Committee of Beijing University of Chinese Medicine (2020BZYLL0805). Written informed consent was obtained from all participants before the study.

### 2.2 Definition of CMM

The primary outcome of this study was CMM, defined as the coexistence of two or more CMDs, including diabetes, heart disease, and stroke (20). Identification of patients with diabetes, heart disease, or stroke was primarily based on self-reporting or current use of corresponding medications such as antidiabetic or cardiovascular drugs. Information on diabetes, heart disease, or stroke was collected through standardized questions: “Have you ever been diagnosed by a doctor with diabetes or high blood sugar?,” “Have you ever been diagnosed by a doctor with coronary heart disease, angina, congestive heart failure, or other heart problems?,” and “Have you ever been diagnosed by a doctor with a stroke?.” Self-reported physician diagnoses have been widely validated in large epidemiological studies and demonstrate good agreement with clinical records (21–24). To minimize recall bias and underreporting, participants who reported any of the above conditions were further asked whether they were currently taking medications or receiving treatment for diabetes, heart disease, or stroke.

### 2.3 Variable selection

#### 2.3.1 Assessment of TCM constitution

Constitutions were assessed using the validated Constitution in Chinese Medicine Questionnaire–Elderly Edition (CCMQ-EE), recommended by the 2013 *Technical Specification for TCM Health Management Services in China* (25). The questionnaire includes 33 items on a 5-point Likert scale and classifies individuals into nine types: one balanced constitution (BC) and eight unbalanced constitutions, including qi-deficiency constitution (QDC), yang-deficiency constitution (YaDC), yin-deficiency constitution (YiDC), phlegm-dampness constitution (PDC), dampness-heat constitution (DHC), blood stasis constitution (BSC), qi stagnation constitution (QSC), and inherited special constitution (ISC). Scores for BC range from 5 to 25 points, while each unbalanced constitution ranges from 4 to 20 points. TCM constitution was determined using the TCM Constitution Batch Identification Tool (Beijing University of Chinese Medicine, Beijing, China) (26), following standardized criteria: (i) BC was assigned if the score ≥17 points, with all unbalanced constitution scores ≤10 points; (ii) If BC < 17 points and an unbalanced constitution score >8 points, that constitution was classified as the principal unbalanced type; (iii) When multiple unbalanced constitutions scored >8 points, the constitution with the highest score was selected; if scores were tied, the constitution with more high-scoring items was chosen as primary.

In terms of psychometric properties, previous studies by our team have reported that the CCMQ demonstrates good internal consistency in Chinese populations, with a Cronbach’s *α* of 0.863 (27). In our study, the CCMQ-EE for older adults showed a Cronbach’s α of 0.885, indicating excellent reliability.

#### 2.3.2 Covariates

The covariates adjusted for in this study included the following categories: (i) Demographics: gender, age, education level (primary school or below, secondary education including junior high, high school, or technical/vocational school, and higher education defined as college diploma or above), occupation, and marital status; (ii) Lifestyle: dietary preferences (bland, spicy, sweet, salty), sleep quality, smoking status (never smoker vs. smoker, including current and former smokers), drinking status (never drinker vs. drinker, including current and former drinkers), and regular physical activity (RPA), defined as frequent participation in activities such as running, Tai Chi, Ba Duan Jin, dancing, gardening, or walking during the past 30 days; (iii) Psychological status: depression and anxiety; (iv) Comorbidities: hyperlipidemia, hypertension, and chronic kidney disease (CKD), all based on self-reported physician diagnoses; (v) Anthropometry: body mass index (BMI), waist circumference (WC), and waist-to-height ratio (WHtR). BMI was calculated as weight (kg) divided by height squared (m^2^) and categorized as underweight (<18.5), normal (18.5–23.9), overweight (24.0–27.9), and obese (≥28.0) (28). Central obesity (CO) was defined as WC ≥ 90 cm in men and ≥85 cm in women (28). WHtR was defined as <0.5 (normal) or ≥0.5 (abnormal) (29).

Sleep quality was evaluated using the Pittsburgh Sleep Quality Index (PSQI), with total scores ranging from 0 to 21; a score >5 indicated poor sleep quality (30). The Chinese version of the PSQI has demonstrated good internal consistency (Cronbach’s *α* = 0.82–0.83) and test–retest reliability (r = 0.77–0.85) among community-dwelling adults with primary insomnia (31, 32). In this study, the PSQI exhibited acceptable internal consistency, with a Cronbach’s α of 0.777, suggesting satisfactory reliability among older adults in China. Depressive symptoms were assessed using the 9-item Patient Health Questionnaire (PHQ-9) (33), with total scores ranging from 0 to 27. A total score ≥5 was considered indicative of at least mild depressive symptoms. The Chinese version of the PHQ-9 has been validated as a reliable tool for depression screening in the general Chinese population, with a Cronbach’s *α* of approximately 0.86 for the total scale (34). In this study, the Cronbach’s α was 0.856, indicating good internal consistency among older adults. Anxiety symptoms were measured using the 7-item Generalized Anxiety Disorder Scale (GAD-7) (35), with total scores ranging from 0 to 21; a score ≥5 was considered indicative of at least mild anxiety symptoms. The Chinese version of the GAD-7 has shown excellent internal consistency across various Chinese populations (36–39), including patients with epilepsy, medical students, pregnant women, and adolescents, with Cronbach’s *α* values typically exceeding 0.90. In this study, the Cronbach’s α was 0.906, indicating excellent reliability among older adults.

### 2.4 Statistical analysis

Categorical variables are expressed as numbers and percentages and were compared using the Chi-square tests. To investigate relationships among the nine TCM constitutions, tetrachoric correlation analysis was employed. This method is appropriate for dichotomous variables assumed to reflect underlying continuous latent traits. Constitution types were classified as present or absent based on standardized questionnaire assessments. Tetrachoric correlation coefficients estimate the Pearson correlation between latent continuous traits and are preferable to Pearson or phi coefficients when binary variables reflect latent continuity. Correlation matrices were computed using the *tetrachoric* function in the psych package (R, version 4.3.3) via maximum likelihood estimation, and visualized as heatmaps to illustrate associations among the constitutions.

Data mining analyses were conducted using IBM SPSS Modeler (version 18.0). Association rule analysis was employed to identify relationships among variables, expressed in the form *A → B*, where *A* represents the antecedent and *B* the consequent. Three evaluation metrics were applied: support, confidence, and lift. *Support* reflects the probability of co-occurrence of *A* and *B* [Support (*A → B*) = P (A, B)], with higher values indicating greater relevance of the rule. *Confidence* measures the conditional probability of *B* given *A* [Confidence (*A → B*) = P (A, B)/P(A)], and higher confidence values denote more reliable rules. *Lift* quantifies the degree to which the presence of *A* increases the likelihood of *B*, relative to its overall probability [Lift (*A → B*) = P (A, B)/P(A)P(B)]. A lift value >1 indicates a positive association, with larger values signifying stronger correlations between the two variables. In this study, association rules were generated using the following thresholds: minimum support of 10%, minimum confidence of 70%, and a maximum antecedent count of 1. Final rules were evaluated based on these criteria, with particular emphasis on lift, given its robustness in detecting meaningful positive associations.

The association between TCM constitution and CMM was further examined using multivariate logistic regression analysis. Three models were constructed: Model 1, unadjusted; Model 2, adjusted for age, gender, education level, occupation, and marital status; and Model 3, additionally adjusted for lifestyle factors (dietary preferences, sleep quality, smoking, drinking, and RPA), psychological status (depression and anxiety), and comorbidities (hyperlipidemia, hypertension, and CKD). Results were expressed as odds ratios (ORs) with 95% confidence intervals (CIs).

To further evaluate the robustness of the association between TCM constitution and CMM, inverse probability of treatment weighting (IPTW) based on propensity scores was applied. IPTW mitigates confounding while retaining the full sample size. Propensity scores were estimated using a logistic regression model, with TCM constitution as the dependent variable and covariates potentially affecting both constitution and CMM risk included as predictors. Exposed group weights were defined as the inverse of the propensity score, and unexposed group weights as the inverse of one minus the propensity score. Stabilized weights were used to enhance estimation efficiency and reduce variance, improving covariate balance and minimizing potential bias. Covariate balance after IPTW or propensity score matching was assessed using the standardized mean difference (SMD), with SMD < 0.1 considered negligible (40, 41) and SMD < 0.25 deemed acceptable (42). The OR and 95% CI for the TCM constitution and CMM were calculated by a weighted logistic regression model.

Stratified analyses were conducted to examine subgroup differences in the associations between TCM constitution and CMM, with stratification factors including gender, age (60–69/70–79/≥80 years), WHtR (normal/abnormal), CO (yes/no), BMI (underweight/normal/overweight/obese), hyperlipidemia, and hypertension. Interaction terms were tested to evaluate heterogeneity across subgroups. Results were presented in forest plots, displaying ORs and 95% CIs.

All analyses were performed using SPSS software (version 25.0) and R software (version 4.3.3). Two-tailed *p* values <0.05 were considered statistically significant.

## 3 Results

### 3.1 Baseline characteristics of the study population

**Figure 2:**
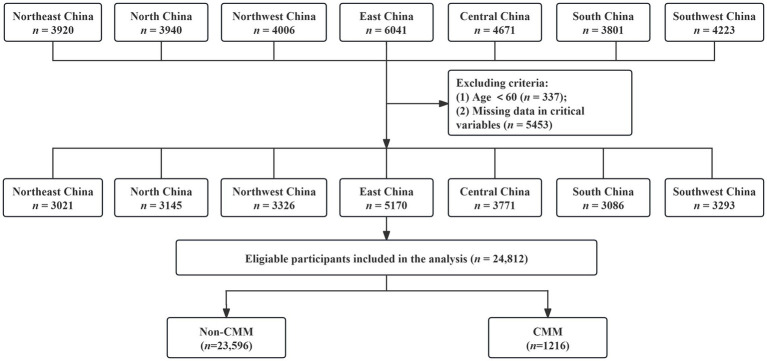
Flow chart of inclusion and exclusion criteria of participants.

As illustrated in the flowchart (Figure 2), a total of 30,602 individuals participated in the survey. Participants younger than 60 years and those with incomplete or invalid questionnaires were excluded. After applying these criteria, 24,812 respondents with complete and valid data were included in the final analysis, yielding an effective response rate of 81.1%.

Among these 24,812 older adults aged 60 years and above, 1,216 (4.9%) were identified as having CMM, comprising 665 females (54.7%) and 551 males (45.3%). Compared with those without CMM, participants with CMM were older (*p* < 0.001), with higher proportions aged 70–79 and 80 years or older, and differed in occupation and marital status (*p* < 0.001). They also exhibited a higher prevalence of CO, abnormal WHtR, overweight, and obesity (*p* < 0.001), and were more likely to smoke or drink, prefer sweet or salty foods, and less likely to favor a bland diet (*p* < 0.05). Additionally, they reported poor sleep quality, higher levels of depression and anxiety, and engaged in regular physical activity (*p* < 0.001). Clinically, hyperlipidemia, hypertension, and CKD were more prevalent in the CMM group (*p* < 0.001) (Table 1).

**Table 1:** Baseline characteristics of participants with and without CMM.

Characteristics	Total (*n* = 24,812)	CMM (*n* = 1,216)	Non-CMM (*n* = 23,596)	χ^2^	*p* value
Gender, *n* (%)				1.075	0.300
Female	13,926 (56.1)	665 (54.7)	13,261 (56.2)		
Male	10,886 (43.9)	551 (45.3)	10,335 (43.8)		
Age, *n* (%)				177.404	<0.001
60–69	14,103 (56.8)	467 (38.4)	13,636 (57.8)		
70–79	7,977 (32.1)	563 (46.3)	7,414 (31.4)		
≥80	2,732 (11.0)	186 (15.3)	2,546 (10.8)		
Education level, *n* (%)				1.818	0.403
Primary education	10,189 (41.1)	497 (40.9)	9,692 (41.1)		
Secondary education	11,842 (47.7)	596 (49.0)	11,246 (47.7)		
Tertiary education	2,781 (11.2)	123 (10.1)	2,658 (11.3)		
Occupation, *n* (%)				29.900	<0.001
Service personnel	1,584 (6.4)	66 (5.4)	1,518 (6.4)		
Workers	9,416 (37.9)	466 (38.3)	8,950 (37.9)		
Farmers	7,717 (31.1)	325 (26.7)	7,392 (31.3)		
Employees and cadres in enterprises and institutions	3,014 (12.1)	178 (14.6)	2,836 (12.0)		
Professional and technical personnel	2,377 (9.6)	126 (10.4)	2,251 (9.5)		
Other occupations	704 (2.8)	55 (4.5)	649 (2.8)		
Marital status, *n* (%)				37.017	<0.001
Married	21,460 (86.5)	981 (80.7)	20,479 (86.8)		
Unmarried	3,352 (13.5)	235 (19.3)	3,117 (13.2)		
CO, *n* (%)				33.662	<0.001
Yes	8,322 (33.5)	501 (41.2)	7,821 (33.1)		
No	16,490 (66.5)	715 (58.8)	15,775 (66.9)		
WHtR, *n* (%)				44.958	<0.001
Normal	10,143 (40.9)	385 (31.7)	9,758 (41.4)		
Abnormal	14,669 (59.1)	831 (68.3)	13,838 (58.6)		
BMI, *n* (%)				46.840	<0.001
<18.5	921 (3.7)	34 (2.8)	887 (3.8)		
18.5 ≤ BMI<24	13,040 (52.6)	545 (44.8)	12,495 (53.0)		
24 ≤ BMI<28	8,586 (34.6)	479 (39.4)	8,107 (34.4)		
≥28	2,265 (9.1)	158 (13.0)	2,107 (8.9)		
Smoking, *n* (%)				35.700	<0.001
Yes	3,374 (13.6)	235 (19.3)	3,139 (13.3)		
No	21,438 (86.4)	981 (80.7)	20,457 (86.7)		
Drinking, *n* (%)				19.664	<0.001
Yes	2,837 (11.4)	187 (15.4)	2,650 (11.2)		
No	21,975 (88.6)	1,029 (84.6)	20,946 (88.8)		
Bland taste, *n* (%)				11.915	0.001
Yes	18,625 (75.1)	862 (70.9)	17,763 (75.3)		
No	6,187 (24.9)	354 (29.1)	5,833 (24.7)		
Spicy taste, *n* (%)				3.017	0.082
Yes	3,005 (12.1)	128 (10.5)	2,877 (12.2)		
No	21,807 (87.9)	1,088 (89.5)	20,179 (87.8)		
Sweet taste, *n* (%)				20.218	<0.001
Yes	2,807 (11.3)	186 (15.3)	2,621 (11.1)		
No	22,005 (88.7)	1,030 (84.7)	20,975 (88.9)		
Salty taste, *n* (%)				25.337	<0.001
Yes	3,498 (14.1)	231 (19.0)	3,267 (13.8)		
No	21,314 (85.9)	985 (81.0)	20,329 (86.2)		
RPA, *n* (%)				16.788	<0.001
Yes	5,133 (20.7)	308 (25.3)	4,825 (20.4)		
No	19,679 (79.3)	908 (74.7)	18,771 (79.6)		
Sleep quality, *n* (%)				117.937	<0.001
Good	13,744 (55.4)	490 (40.3)	13,254 (56.2)		
Poor	11,068 (44.6)	726 (59.7)	10,342 (43.8)		
Depression, *n* (%)				127.255	<0.001
Yes	4,853 (19.6)	390 (32.1)	4,463 (18.9)		
No	19,959 (80.4)	826 (67.9)	19,133 (81.1)		
Anxiety, *n* (%)				35.095	<0.001
Yes	3,194 (12.9)	224 (18.4)	2,970 (12.6)		
No	21,618 (87.1)	992 (81.6)	20,626 (87.4)		
Hyperlipidemia, *n* (%)				343.801	<0.001
Yes	1,597 (6.4)	233 (19.2)	1,364 (5.8)		
No	23,215 (93.6)	983 (80.8)	22,232 (94.2)		
Hypertension, *n* (%)				586.628	<0.001
Yes	8,744 (35.2)	822 (67.6)	7,922 (33.6)		
No	16,068 (64.8)	394 (32.4)	15,674 (66.4)		
CKD, *n* (%)				52.265	<0.001
Yes	252 (1.0)	37 (3.0)	215 (0.9)		
No	24,560 (99.0)	1,179 (97.0)	23,381 (99.1)		

CO, Central Obesity; WHtR, waist-to-height ratio; BMI, body Mass Index; RPA, regular physical activity; CKD, chronic kidney disease.

### 3.2 Distribution characteristics of TCM constitution in older adults with CMM

**Figure 3:**
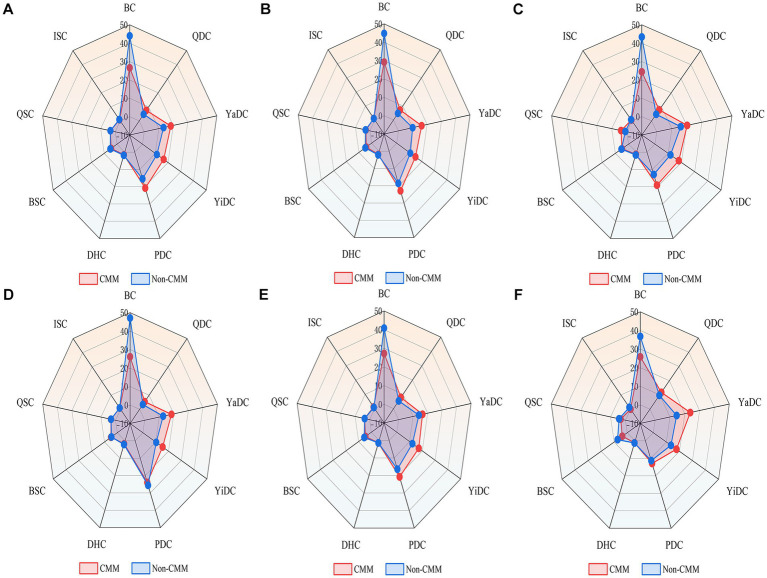
Radar plots of TCM constitution distribution in older adults with and without CMM. Light red represents the CMM group, and light blue represents the non-CMM group. **(A)** Overall population; **(B)** Male participants; **(C)** Female participants; **(D)** Participants aged 60–69 years; **(E)** Participants aged 70–79 years; **(F)** Participants aged ≥80 years. BC, balanced constitution; QDC, qi-deficiency constitution; YaDC, yang-deficiency constitution; YiDC, yin-deficiency constitution; PDC, phlegm-dampness constitution; DHC, dampness-heat constitution; BSC, blood stasis constitution; QSC, qi stagnation constitution; ISC, inherited special constitution.

Regarding the distribution of TCM constitution types, the BC was the most common type overall (43.2%), but its prevalence was significantly lower among individuals with CMM across all subgroups. In contrast, QDC, YaDC, YiDC, and PDC were observed at significantly higher proportions in the CMM group (*p* < 0.001), whereas no significant differences were found for the other constitution types (Table 2). Stratified analyses showed that these differences were present in both men and women, with significant group differences detected for BC, QDC, YaDC, YiDC, and PDC in each gender (Supplementary Tables S1, S2). Age-specific analyses further indicated that group differences in QDC, YiDC, and PDC were observed in the 60–79 years cohort, while in participants aged ≥80 years, only YaDC showed a significant difference (Supplementary Tables S3–S5). Overall, the distribution of TCM constitutions differed significantly between older adults with and without CMM, with consistently lower frequencies of BC and higher frequencies of QDC, YaDC, YiDC, and PDC in the CMM group (Figure 3).

**Table 2:** Distribution of TCM constitution in older adults with and without CMM.

TCM constitution	Total	CMM		χ^2^	*p*-value
		Yes (*n* = 1,216)	No (*n* = 23,596)		
BC, *n* (%)				143.594	<0.001
Yes	10,709 (43.2)	323 (26.6)	10,386 (44.0)		
No	14,103 (56.8)	893 (73.4)	13,210 (56.0)		
QDC, *n* (%)				19.618	<0.001
Yes	1,198 (4.8)	91 (7.5)	1,107 (4.7)		
No	23,614 (95.2)	1,125 (92.5)	22,489 (95.3)		
YaDC, *n* (%)				25.277	<0.001
Yes	3,357 (13.5)	223 (18.3)	3,134 (13.3)		
No	21,455 (86.5)	993 (81.7)	20,462 (86.7)		
YiDC, *n* (%)				31.633	<0.001
Yes	2,873 (11.6)	202 (16.6)	2,671 (11.3)		
No	21,939 (88.4)	1,014 (83.4)	20,925 (88.7)		
PDC, *n* (%)				25.662	<0.001
Yes	3,885 (15.7)	253 (20.8)	3,632 (15.4)		
No	20,927 (84.3)	963 (79.2)	19,964 (84.6)		
DHC, *n* (%)				0.729	0.393
Yes	446 (1.8)	18 (1.5)	428 (1.8)		
No	24,366 (98.2)	1,198 (98.5)	23,168 (98.2)		
BSC, *n* (%)				0.823	0.364
Yes	1,282 (5.2)	56 (4.6)	1,226 (5.2)		
No	23,530 (94.8)	1,160 (95.4)	22,370 (94.8)		
QSC, *n* (%)				0.008	0.931
Yes	827 (3.3)	40 (3.3)	787 (3.3)		
No	23,985 (96.7)	1,176 (96.7)	22,809 (96.7)		
ISC, *n* (%)				0.212	0.645
Yes	235 (0.9)	10 (0.8)	225 (1.0)		
No	24,577 (99.1)	1,206 (99.2)	23,371 (99.0)		

BC, balanced constitution; QDC, qi-deficiency constitution; YaDC, yang-deficiency constitution; YiDC, yin-deficiency constitution; PDC, phlegm-dampness constitution; DHC, dampness-heat constitution; BSC, blood stasis constitution; QSC, qi stagnation constitution; ISC, inherited special constitution.

**Figure 4:**
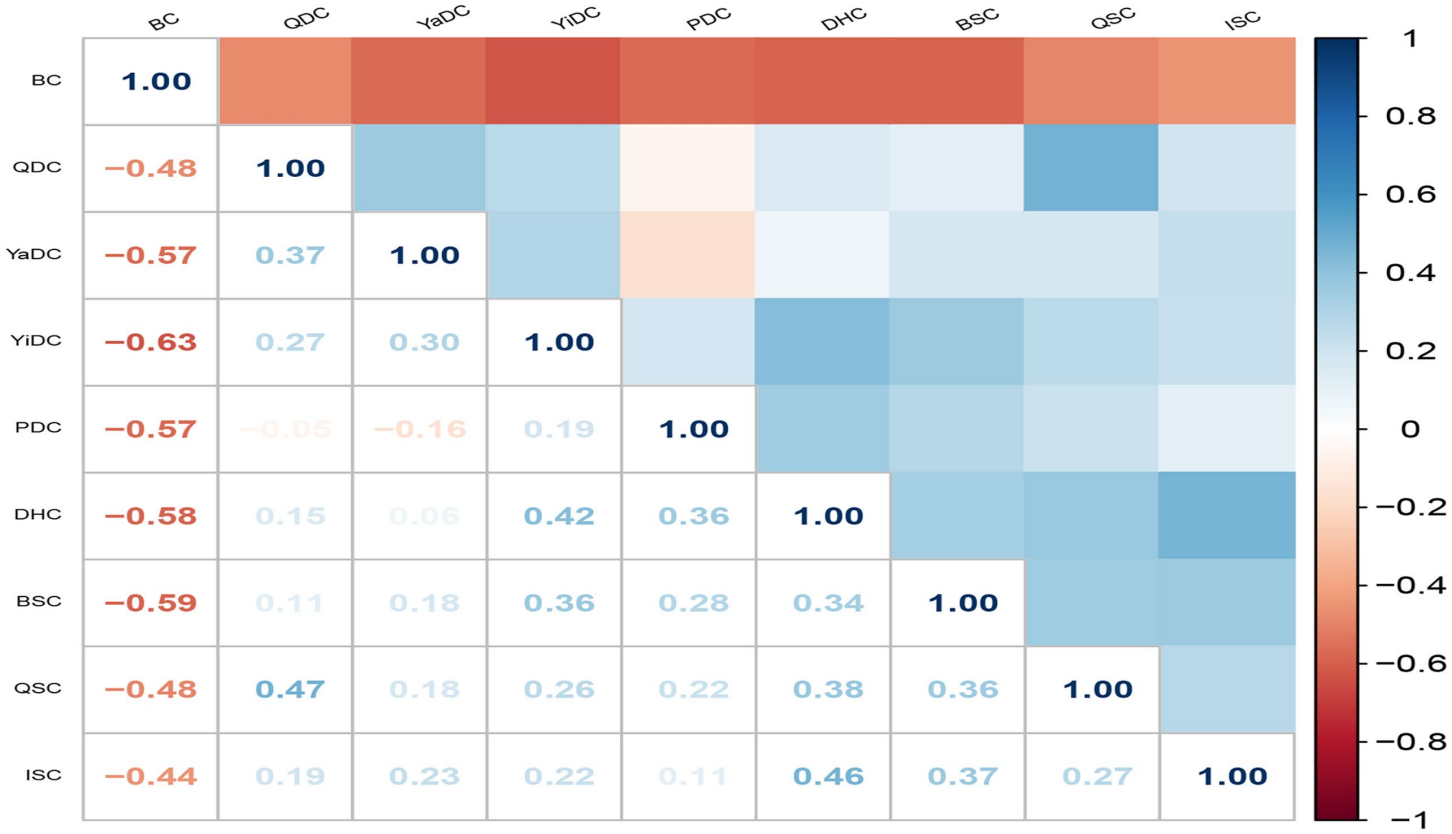
Tetrachoric correlation matrix of the nine TCM constitutions in 1216 older adults with CMM. Color represents the tetrachoric correlation coefficient between TCM constitutions. Darker blue indicates a stronger positive correlation, while darker red indicates a stronger negative correlation. BC, balanced constitution; QDC, qi-deficiency constitution; YaDC, yang-deficiency constitution; YiDC, yin-deficiency constitution; PDC, phlegm-dampness constitution; DHC, dampness-heat constitution; BSC, blood stasis constitution; QSC, qi stagnation constitution; ISC, inherited special constitution.

We further explored whether specific constitution types tend to coexist or cluster among older adults with CMM, aiming to clarify potential interrelationships between TCM constitutions. Among 1,216 patients with CMM, 12.2% exhibited BC, whereas 87.8% had unbalanced constitutions, 90.7% of which were mixed. As shown in Figure 4, BC was negatively associated with all unbalanced constitution types, strongly with YiDC and moderately with YaDC, PDC, DHC, and BSC. Moderate positive correlations (r > 0.35) were observed among unbalanced constitutions, including QDC with QSC and YaDC; YiDC with DHC and BSC; PDC with DHC; DHC with QSC and ISC; and BSC with QSC and ISC. Association rule analysis identified three constitution pairings with strong co-occurrence, with DHC and YiDC showing the highest confidence, followed by BSC and YiDC, and DHC and PDC (Table 3).

**Table 3:** Association rules of nine TCM constitutions in CMM population.

Consequent	Antecedent	Support (%)	Confidence (%)	Lift	Number
YiDC	DHC	19.33	78.30	1.43	235
YiDC	BSC	32.48	70.89	1.29	395
PDC	DHC	19.33	70.21	1.41	235

YiDC, yin-deficiency constitution; PDC, phlegm-dampness constitution; DHC, dampness-heat constitution; BSC, blood stasis constitution.

### 3.3 Association between TCM constitution type and CMM

The association between TCM constitutions and CMM was analyzed using multivariate logistic regression models, with BC as the reference group. In the unadjusted model, a significant positive association between TCM constitutions and CMM was observed: QDC [OR (95% CI) = 2.643 (2.078–3.363), *p* < 0.001], YaDC [OR (95% CI) = 2.288 (1.920–2.726), *p* < 0.001], YiDC [OR (95% CI) = 2.432 (2.029–2.914), *p* < 0.001], PDC [OR (95% CI) = 2.240 (1.892–2.652), *p* < 0.001], BSC [OR (95% CI) = 1.469 (1.099–1.962), *p* = 0009] and QSC [OR (95% CI) = 1.634 (1.167–2.288), *p* = 0.004]. After partial adjustment for demographic information, QDC, YaDC, YiDC, PDC, and BSC remained significantly associated with CMM: QDC [OR (95% CI) = 2.337 (1.830–2.984), *p* < 0.001], YaDC [OR (95% CI) = 2.167 (1.851–2.587), *p* < 0.001], YiDC [OR (95% CI) = 2.312 (1.926–2.775), *p* < 0.001], PDC [OR (95% CI) = 2.167 (1.828–2.569), *p* < 0.001], BSC [OR (95% CI) = 1.347 (1.006–1.804), *p* = 0.046], and QSC [OR (95% CI) = 1.596 (1.138–2.238), *p* = 0.007]. In the fully adjusted model, which accounted for demographic information, lifestyle habits, psychological condition, and comorbidities, QDC, YaDC, YiDC, and PDC still showed significant associations with CMM: QDC [OR (95% CI) = 1.570 (1.209–2.038), *p* = 0.001], YaDC [OR (95% CI) = 1.625 (1.346–1.961), *p* < 0.001], YiDC [OR (95% CI) = 1.617 (1.333–1.962), *p* < 0.001], and PDC [OR (95% CI) = 1.403 (1.173–1.678), *p* < 0.001]. No significant association was found between other types of TCM constitution and CMM in the fully adjusted model (Table 4).

**Table 4:** Relationship between TCM constitution and CMM in different models.

TCM constitution	Model 1		Model 2		Model 3	
	OR (95% CI)	*p*	OR (95% CI)	*p*	OR (95% CI)	*p*
BC	*Ref*					
QDC	2.643 (2.078 ~ 3.363)	<0.001	2.337 (1.830 ~ 2.984)	<0.001	1.570 (1.209 ~ 2.038)	0.001
YaDC	2.288 (1.920 ~ 2.726)	<0.001	2.167 (1.815 ~ 2.587)	<0.001	1.625 (1.346 ~ 1.961)	<0.001
YiDC	2.432 (2.029 ~ 2.914)	<0.001	2.312 (1.926 ~ 2.775)	<0.001	1.617 (1.333 ~ 1.962)	<0.001
PDC	2.240 (1.892 ~ 2.652)	<0.001	2.167 (1.828 ~ 2.569)	<0.001	1.403 (1.173 ~ 1.678)	<0.001
DHC	1.352 (0.833 ~ 2.195)	0.222	1.392 (0.855 ~ 2.264)	0.183	0.952 (0.729 ~ 1.339)	0.848
BSC	1.469 (1.099 ~ 1.962)	0.009	1.347 (1.006 ~ 1.804)	0.046	0.988 (0.729–1.339)	0.938
QSC	1.634 (1.167 ~ 2.288)	0.004	1.596 (1.138 ~ 2.238)	0.007	1.061 (0.735 ~ 1.530)	0.753
ISC	1.429 (0.751 ~ 2.718)	0.276	1.398 (0.733 ~ 2.668)	0.309	1.070 (0.552 ~ 2.074)	0.842

Model 1 was unadjusted. Model 2 was adjusted for gender, age, education level, occupation, and marital status. Model 3 was Model 2 further adjusted for lifestyle habits (smoking, drinking, dietary preferences, sleep quality, and RPA), psychological conditions (depression and anxiety), and comorbidities (hypertension, hyperlipidemia, and CKD). BC, balanced constitution; QDC, qi-deficiency constitution; YaDC, yang-deficiency constitution; YiDC, yin-deficiency constitution; PDC, phlegm-dampness constitution; DHC, dampness-heat constitution; BSC, blood stasis constitution; QSC, qi stagnation constitution; ISC, inherited special constitution.

### 3.4 Sensitivity analysis

**Figure 5:**
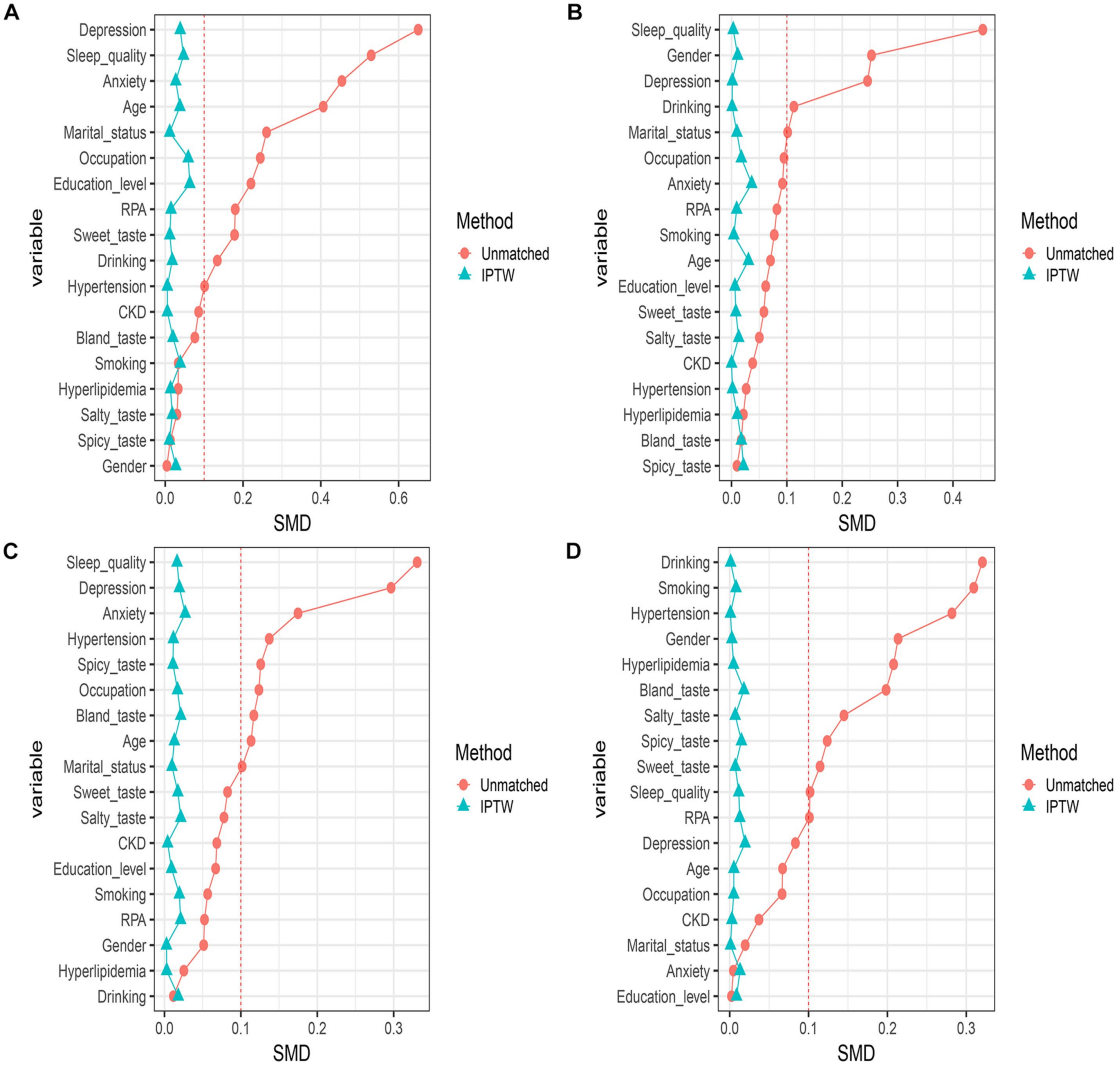
Balance diagnostics after inverse probability weighting of propensity scores. **(A)** Balance diagnostic after inverse probability weighting of QDC propensity scores. **(B)** Balance diagnostics after inverse probability weighting of YaDC propensity scores. **(C)** Balance diagnostics after inverse probability weighting of YiDC propensity scores. **(D)** Balance diagnostics after inverse probability weighting of PDC propensity scores. RPA, regular physical activity; CKD, chronic kidney disease.

To verify the robustness of the relationship between TCM constitution and CMM, we used the IPTW based on propensity scores. Participants were grouped according to whether they were QDC, YaDC, YiDC, or PDC. Covariates were then controlled using stabilized IPTW calculated based on propensity scores, including confounding factors: demographic information, lifestyle habits, psychological condition, and comorbidities. The SMD of matched confounders was less than 0.1 for all groups (QDC vs. non-QDC, YaDC vs. non-YaDC, YiDC vs. non-YiDC, and PDC vs. non-PDC), indicating that covariates were well-balanced after matching (Supplementary Tables S6–S9; Figures 5A–D). Subsequently, the weighted logistic regression model was used to estimate the OR with 95% CI for the association of each group with CMM. The results were as follows: QDC [1.539 (1.422–1.665)], YaDC [1.338 (1.234–1.450)], YiDC [1.290 (1.190–1.399)], and PDC [1.152 (1.061–1.252)] (Tables 5–8). After IPTW based on propensity score analysis, QDC, YaDC, YiDC and PDC remained positively associated with CMM.

**Table 5:** Association of the QDC with CMM after IPTW matching.

After IPTW	QDC		OR	
	No	Yes	OR (95% CI)	*p*
CMM, *n* (%)	1,202 (4.8)	1,694 (7.0)	1.539 (1.422 ~ 1.665)	<0.001

QDC, qi-deficiency constitution; CMM, Cardiometabolic multimorbidity; OR, odds ratios; CI, confidence intervals.

**Table 6:** Association of the YaDC with CMM after IPTW matching.

After IPTW	YaDC		OR	
	No	Yes	OR (95% CI)	*p*
CMM, *n* (%)	1,172 (4.7)	1,509 (6.1)	1.338 (1.234 ~ 1.450)	<0.001

YaDC, yang-deficiency constitution; CMM, Cardiometabolic multimorbidity; OR, odds ratios; CI, confidence intervals.

**Table 7:** Association of the YiDC with CMM after IPTW matching.

After IPTW	YiDC		OR	
	No	Yes	OR (95% CI)	*p*
CMM, *n* (%)	1,180 (4.8)	1,470 (6.0)	1.290 (1.190 ~ 1.399)	<0.001

YiDC, yin-deficiency constitution; CMM, Cardiometabolic multimorbidity; OR, odds ratios; CI, confidence intervals.

**Table 8:** Association of the PDC with CMM after IPTW matching.

After IPTW	PDC		OR	
	No	Yes	OR (95% CI)	*p*
CMM, *n* (%)	1,193 (4.8)	1,330 (5.3)	1.152 (1.061 ~ 1.252)	<0.001

PDC, phlegm-dampness constitution; CMM, Cardiometabolic multimorbidity; OR, odds ratios; CI, confidence intervals.

### 3.5 Subgroup analysis

**Figure 6:**
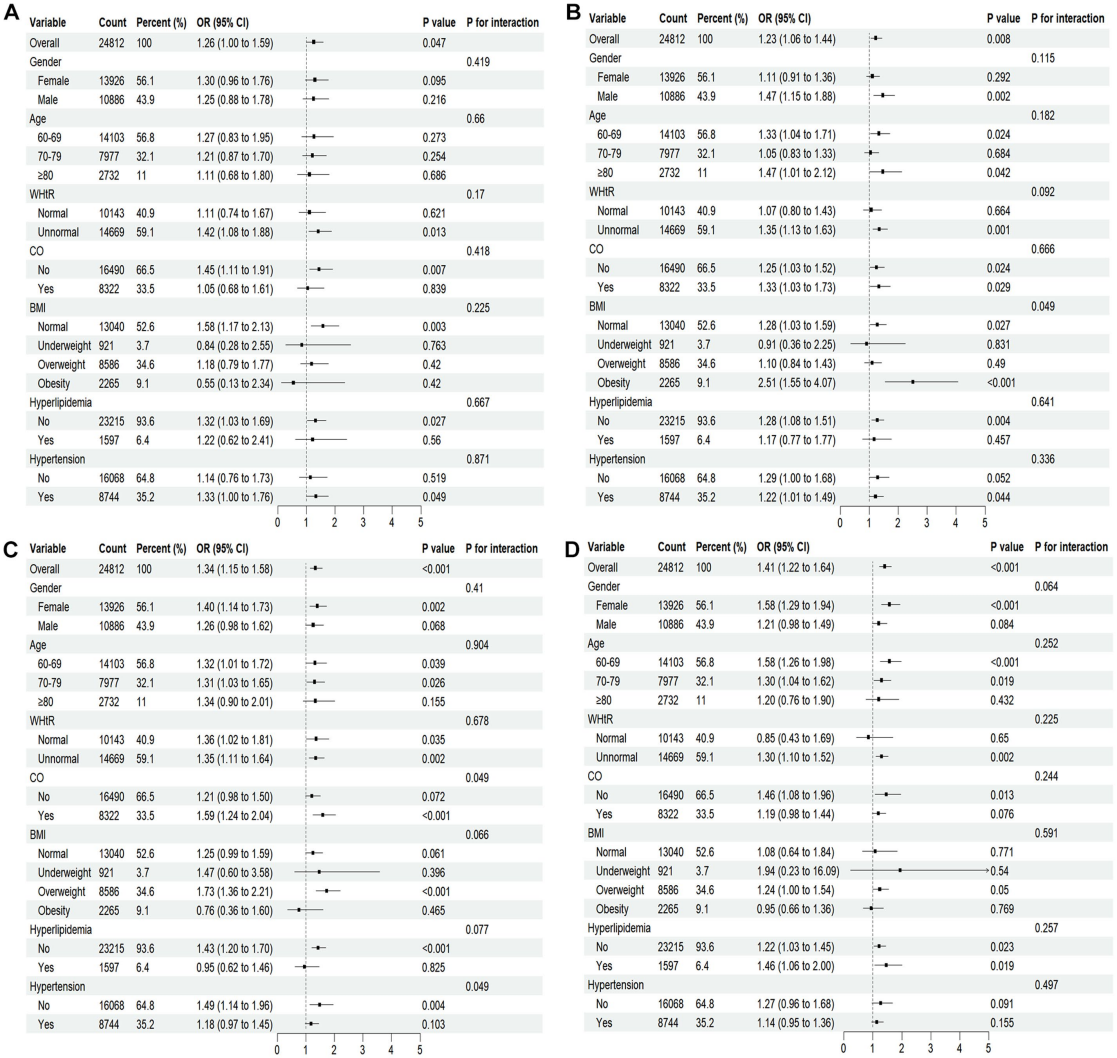
Forest plots of stratified analyses of TCM constitution and CMM. Fully adjusted model: adjusted for all stratification factors plus education level, occupation, marital status, smoking, drinking, dietary preferences, sleep quality, RPA, depression, anxiety, and CKD. **(A–D)** Represent subgroups with qi-deficiency, yang-deficiency, yin-deficiency, and phlegm-dampness constitutions, respectively. WHtR, waist-to-height ratio; CO, central obesity; BMI, body mass index.

Subgroup analyses were performed to assess potential effect modifications by sex, age, WHtR, CO, BMI, hyperlipidemia, and hypertension on the associations between TCM constitutions (QDC, YaDC, YiDC, and PDC) and CMM (Supplementary Figure S1; Figure 6). In fully adjusted models, the associations of QDC and PDC with CMM remained generally consistent across all subgroups, with no significant interactions observed. In contrast, significant effect modifications were identified for YaDC and YiDC. Specifically, the association between YaDC and CMM was stronger among individuals with obesity (*P* for interaction = 0.049). Similarly, the association of YiDC with CMM was more pronounced in participants with CO and in those without hypertension (both *P* for interaction = 0.049) (Figure 6).

## 4 Discussion

This study, based on a large, nationally representative sample of older Chinese adults, is the first to comprehensively explore the relationship between TCM constitution types and CMM. Four unbalanced constitution types—QDC, YaDC, YiDC, and PDC—were independently and consistently associated with increased risk of CMM. These associations remained robust after extensive covariate adjustment and IPTW based on propensity scores, supporting their stability and internal validity. Subgroup analyses revealed distinct effect modification patterns: the association between YaDC and CMM was stronger among individuals with obesity, while YiDC showed a greater impact among those with central obesity and without hypertension. Moreover, most older adults with CMM displayed mixed constitutions, indicating complex constitution interrelations in multimorbid populations.

Our findings revealed a positive association between QDC and CMM in older adults, with no significant effect modification by sex, age, obesity indices, or comorbid conditions. In TCM theory, QDC denotes a state of diminished vital energy, typically manifested as fatigue, shortness of breath, and reduced immune resilience (11). Biomedically, this constitutional profile parallels reduced cardiorespiratory fitness, sarcopenia, and impaired metabolic homeostasis (43), all of which are independent predictors of cardiometabolic disorders (44–46). The generally consistent association observed across diverse subgroups suggests that Qi-deficiency may constitute a fundamental physiological vulnerability in aging populations, predisposing individuals to cumulative metabolic dysregulation. These findings highlight that interventions aimed at enhancing vitality and metabolic efficiency, such as exercise-based rehabilitation or Qi-tonifying herbal therapies, may have broad preventive potential for mitigating CMM risk among older adults.

Beyond QDC, YaDC was also positively associated with CMM, with stronger associations observed among obese individuals (P for interaction = 0.049). YaDC is characterized by cold intolerance, diminished metabolic activity, and sluggish circulation (47). Prior evidence indicates that YaDC is related to reduced basal metabolic rate, impaired thermogenesis, and increased adiposity (48, 49), which could partly account for the amplified CMM risk observed in individuals with obesity. The significant interaction suggests that obesity may exacerbate the adverse metabolic consequences of YaDC, reinforcing the need for constitution-specific risk stratification. Interventions focused on warming Yang, enhancing energy metabolism, and improving circulatory function may be particularly beneficial for obese older adults with YaDC, potentially mitigating their elevated susceptibility to CMM.

YiDC was significantly associated with CMM, with stronger associations observed in participants with central obesity and in those without hypertension (P for interaction = 0.049). In TCM theory, YiDC is characterized by internal heat, fluid depletion, and dryness (11). Insufficient Yin fails to effectively restrain Yang, resulting in pathological “deficiency heat,” which manifests as restlessness, dry mouth, and heightened metabolic activity, predisposing to vascular injury. This features align with biomedical evidence linking YiDC to systemic low-grade inflammation (50), oxidative stress (51), and insulin resistance (52). The stronger association identified in centrally obese individuals may reflect synergistic effects of adiposity-related heat and metabolic stress exacerbating Yin-deficiency manifestations. Notably, the more pronounced association in those without hypertension suggests that YiDC may promote CMM primarily through non-hypertensive pathways, such as dyslipidemia, impaired glucose regulation, and endothelial dysfunction. Overall, these findings indicate that YiDC may represent a constitution type particularly vulnerable to CMM development, especially in the presence of central obesity, and that interventions aimed at nourishing Yin and restoring fluid balance may offer preventive benefits in this subgroup.

PDC was robustly associated with CMM, with generally consistent effects across all examined subgroups. In TCM theory, PDC is characterized by heaviness, sluggishness, and susceptibility to obesity and metabolic stagnation (11, 53). Previous studies have demonstrated that PDC is closely associated with obesity, insulin resistance, and dyslipidemia (43)—established risk factors for cardiometabolic disorders. These findings suggest that PDC reflects a clustering of metabolic abnormalities predisposing individuals to CMM. Given its stability across sex, age, obesity, and major metabolic risk factors, PDC may serve as an accessible, non-invasive marker for identifying individuals at elevated CMM risk, particularly in resource-limited settings where biochemical testing may not be readily available. Interventions that target weight reduction, improvement of metabolic clearance, and resolution of low-grade inflammation—whether through lifestyle modification, pharmacotherapy, or TCM-based therapies aimed at resolving phlegm and dampness—may hold particular promise for older adults with this constitution type.

A high prevalence of mixed constitutions among older adults with CMM aligns with TCM theory, which posits that aging involves the decline of both Yin and Yang, insufficiency of Qi and Blood, and progressive organ weakening (54). The positive correlations observed among unbalanced constitution types further indicated that constitutions rarely exist in isolation in multimorbid populations. Notably, the frequent coexistence of YiDC with DHC and BSC, or PDC with DHC, may reflect shared metabolic and inflammatory mechanisms. For example, YiDC is associated with internal heat and fluid depletion, which may promote damp-heat accumulation and vascular stasis, thereby increasing the risk of atherosclerosis and insulin resistance. Similarly, the coexistence of PDC and DHC suggests overlapping mechanisms of metabolic dysregulation. PDC is characterized by impaired fluid metabolism, obesity, and accumulation of turbidity, while DHC represents the combination of excessive dampness with internal heat, often leading to systemic inflammation (55, 56). When present together, these constitutions may act synergistically to exacerbate lipid metabolism disorders, endothelial dysfunction, and chronic low-grade inflammation, thereby amplifying susceptibility to CMM.

In this study, all measurement instruments, including the CCMQ-EE, PHQ-9, GAD-7, and PSQI, demonstrated good to excellent internal consistency reliability, with Cronbach’s *α* values ranging from 0.777 to 0.906. These findings confirm that the instruments are psychometrically robust and appropriate for assessing TCM constitution, sleep quality, depressive symptoms, and anxiety symptoms among community-dwelling older adults in China. The high reliability of these scales strengthens the confidence in the observed associations between TCM constitution and CMM, supporting the validity of our findings.

From a clinical perspective, these findings underscore the potential value of incorporating TCM constitution assessment into the prevention and management of CMM. Identification of constitution types may facilitate stratification of older adults into higher- and lower-risk subgroups, complementing conventional risk prediction tools. Furthermore, constitution-guided interventions—such as individualized herbal prescriptions, acupuncture, or lifestyle modifications tailored to each constitution type—may provide personalized strategies to reduce the burden of multimorbidity. Compared with conventional management approaches, which are often limited by disease-specific guidelines and polypharmacy concerns, constitution-informed interventions emphasize targeted, individualized approaches for managing multiple cardiometabolic conditions in older adults.

Several limitations warrant consideration. First, the cross-sectional design precludes causal inference, and longitudinal studies are needed to establish temporal relationships between TCM constitution and CMM development. Second, constitution classification was based on standardized self-report questionnaires rather than clinical diagnosis, which may introduce misclassification bias. Third, although extensive adjustments and propensity score weighting were applied, residual confounding cannot be completely excluded. Fourth, this study focused on examining the association between TCM constitution and CMM in older Chinese adults at the national level, without conducting region-specific analyses. Finally, as this study focused on older Chinese adults, generalizability to other ethnic or cultural populations remains to be determined. Future research should include longitudinal and interventional studies to elucidate causal pathways, and mechanistic investigations integrating constitution assessment with metabolic, inflammatory, and omics-based profiling may further advance personalized, constitution-driven prevention frameworks for CMM. In addition, studies with larger region-specific sample sizes are warranted to explore potential geographic variations in CMM patterns and their associations with TCM constitution, which may inform regionally tailored preventive strategies.

## 5 Conclusion

This nationwide cross-sectional study provides the first robust evidence that QDC, YaDC, YiDC, and PDC are independently associated with CMM in older adults. The frequent coexistence of mixed constitutions, particularly YiDC with DHC or BSC, and PDC with DHC, suggests complex constitution interrelations underlying multimorbidity. These findings underscore the potential of TCM constitution assessment as a complementary approach for early risk identification and constitution-targeted prevention. Integrating constitution-based management into routine care may help reduce susceptibility to CMM and improve outcomes in older adults. Further longitudinal and interventional studies are warranted to clarify causal mechanisms and validate constitution-focused strategies.

## Data Availability

The raw data supporting the conclusions of this article will be made available by the authors, without undue reservation.
