# Supplementary material for: Traditional Chinese medicine constitution and cardiometabolic multimorbidity: a nationwide cross-sectional study in older adults
Source: Front Public Health. 2026 Jan 26;14:1723708. doi: 10.3389/fpubh.2026.1723708 (PMC12883366; doi:10.3389/fpubh.2026.1723708)
Supplement: Supplementary file 1 [file Table_1.docx]

Supplementary Material

# **1 Supplementary Figures**

**
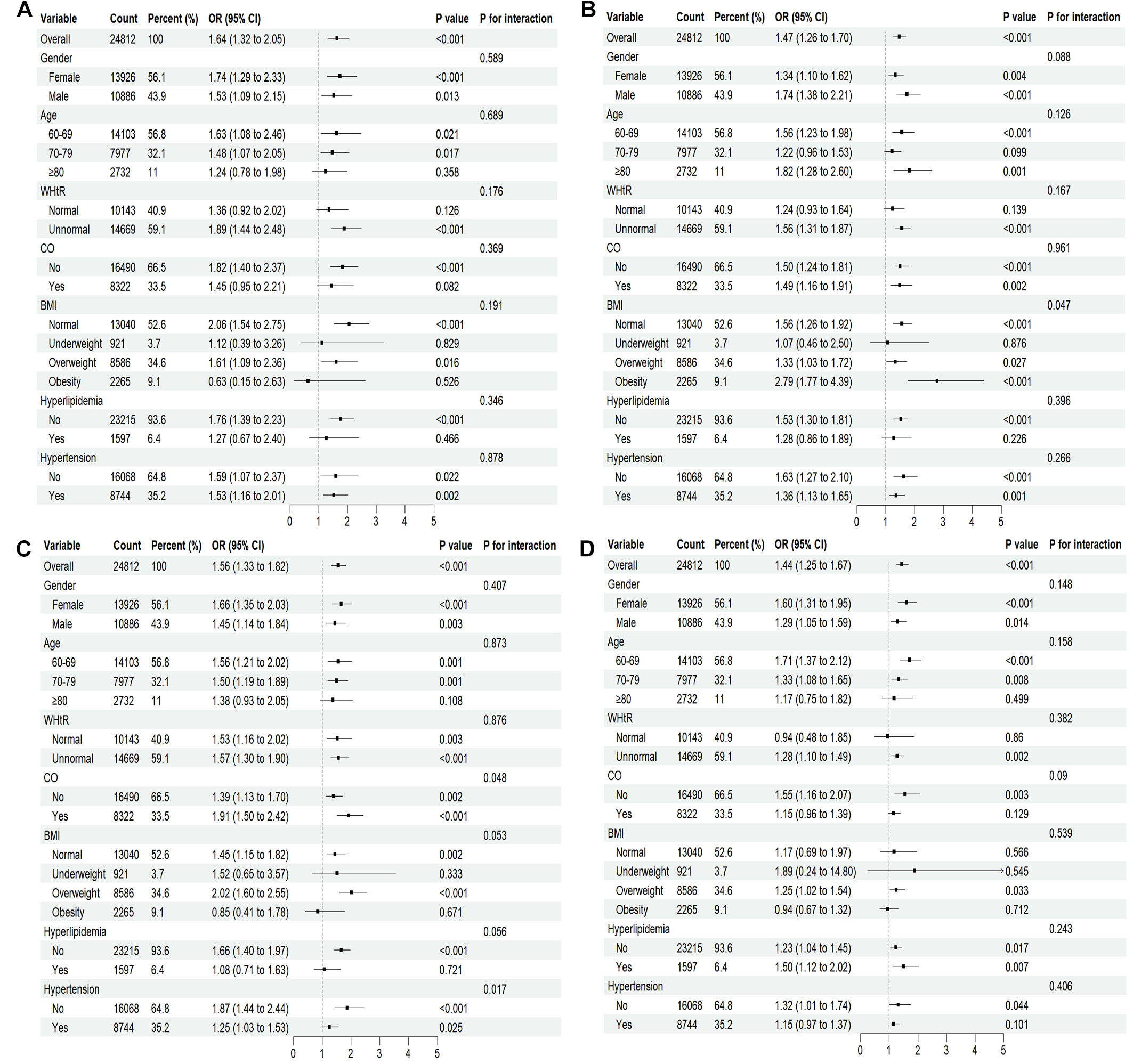
**

**Supplementary Figure S1** Forest plots of stratified analyses of TCM constitution and CMM (unadjusted model). (**A–D**) Represent subgroups with qi-deficiency, yang-deficiency, yin-deficiency, and phlegm-dampness constitutions, respectively. WHtR, waist-to-height ratio; CO, central obesity; BMI, body mass index.

# **2 Supplementary Tables**

**Supplementary Table S1** Distribution of TCM constitutions in older male adults with and without CMM.

| TCM constitution | Total  (n=10886) | CMM | | χ^2^ | *P*-value |
| --- | --- | --- | --- | --- | --- |
|  |  | Yes (n=551) | No (n=10335) |  |  |
| BC, n (%) | 4792 (44.0) | 161 (29.2) | 4631 (44.8) | 51.590 | ＜0.001 |
| QDC, n (%) | 528 (4.9) | 39 (7.1) | 489 (4.7) | 6.241 | 0.012 |
| YaDC, n (%) | 1117 (10.3) | 89 (16.2) | 1028 (9.9) | 21.878 | ＜0.001 |
| YiDC, n (%) | 1196 (11.0) | 82 (14.9) | 1114 (10.8) | 9.005 | 0.003 |
| PDC, n (%) | 2052 (18.8) | 126 (22.9) | 1926 (18.6) | 6.124 | 0.013 |
| DHC, n (%) | 230 (2.1) | 10 (1.8) | 220 (2.1) | 0.249 | 0.618 |
| BSC, n (%) | 537 (4.9) | 23 (4.2) | 514 (5.0) | 0.712 | 0.399 |
| QSC, n (%) | 323 (3.0) | 15 (2.7) | 308 (3.0) | 0.121 | 0.728 |
| ISC, n (%) | 111 (1.0) | 6 (1.1) | 105 (1.0) | 0.028 | 0.868 |
| χ^2^ | 68.503 | | | | |
| *P*-value | ＜0.001 | | | | |

BC, balanced constitution; QDC, qi-deficiency constitution; YaDC, yang-deficiency constitution; YiDC, yin-deficiency constitution; PDC, phlegm-dampness constitution; DHC, dampness-heat constitution; BSC, blood stasis constitution; QSC, qi stagnation constitution; ISC, inherited special constitution.

**Supplementary Table S2** Distribution of TCM constitutions in older female adults with and without CMM.

| TCM constitution | Total  (n=13926) | CMM | | χ^2^ | *P*-value |
| --- | --- | --- | --- | --- | --- |
|  |  | Yes (n=665) | No (n=13261) |  |  |
| BC, n (%) | 5917 (42.5) | 162 (24.4) | 5755 (43.4) | 93.917 | ＜0.001 |
| QDC, n (%) | 670 (4.8) | 52 (7.8) | 618 (4.7) | 13.801 | ＜0.001 |
| YaDC, n (%) | 2240 (16.1) | 134 (20.2) | 2106 (15.9) | 8.551 | 0.003 |
| YiDC, n (%) | 1677 (12.0) | 120 (18.0) | 1557 (11.7) | 23.758 | ＜0.001 |
| PDC, n (%) | 1833 (13.2) | 127 (19.1) | 1706 (12.9) | 21.524 | ＜0.001 |
| DHC, n (%) | 216 (1.6) | 8 (1.2) | 208 (1.6) | 0.554 | 0.457 |
| BSC, n (%) | 745 (5.3) | 33 (5.0) | 712 (5.4) | 0.207 | 0.649 |
| QSC, n (%) | 504 (3.6) | 25 (3.8) | 479 (3.6) | 0.039 | 0.843 |
| ISC, n (%) | 124 (0.9) | 4 (0.6) | 120 (0.9) | 0.661 | 0.416 |
| χ^2^ | 115.347 | | | | |
| *P*-value | ＜0.001 | | | | |

BC, balanced constitution; QDC, qi-deficiency constitution; YaDC, yang-deficiency constitution; YiDC, yin-deficiency constitution; PDC, phlegm-dampness constitution; DHC, dampness-heat constitution; BSC, blood stasis constitution; QSC, qi stagnation constitution; ISC, inherited special constitution.

**Supplementary Table S3** Distribution of TCM constitutions in older adults aged 60-69 years with and without CMM.

| TCM constitution | Total  (n=14103) | CMM | | χ^2^ | *P*-value |
| --- | --- | --- | --- | --- | --- |
|  |  | Yes (n=467) | No (n=13636) |  |  |
| BC, n (%) | 6548 (46.4) | 122 (26.1) | 6426 (47.1) | 80.067 | ＜0.001 |
| QDC, n (%) | 483 (3.4) | 25 (5.4) | 458 (3.4) | 5.431 | 0.020 |
| YaDC, n (%) | 1812 (12.8) | 86 (18.4) | 1726 (12.7) | 13.368 | ＜0.001 |
| YiDC, n (%) | 1496 (10.6) | 72 (15.4) | 1424 (10.4) | 11.784 | 0.001 |
| PDC, n (%) | 2238 (15.9) | 112 (24.0) | 2126 (15.6) | 23.818 | ＜0.001 |
| DHC, n (%) | 299 (2.1) | 8 (1.7) | 291 (2.1) | 0.386 | 0.535 |
| BSC, n (%) | 645 (4.6) | 23 (4.9) | 622 (4.6) | 0.137 | 0.712 |
| QSC, n (%) | 442 (3.1) | 15 (3.2) | 427 (3.1) | 0.010 | 0.922 |
| ISC, n (%) | 140 (1.0) | 4 (0.9) | 136 (1.0) | 0.091 | 0.763 |
| χ^2^ | 90.967 | | | | |
| *P*-value | ＜0.001 | | | | |

BC, balanced constitution; QDC, qi-deficiency constitution; YaDC, yang-deficiency constitution; YiDC, yin-deficiency constitution; PDC, phlegm-dampness constitution; DHC, dampness-heat constitution; BSC, blood stasis constitution; QSC, qi stagnation constitution; ISC, inherited special constitution.

**Supplementary Table S4** Distribution of TCM constitutions in older adults aged 70-79 years with and without CMM.

| TCM constitution | Total  (n=7977) | CMM | | χ^2^ | *P*-value |
| --- | --- | --- | --- | --- | --- |
|  |  | Yes (n=563) | No (n=7414) |  |  |
| BC, n (%) | 3177 (39.8) | 153 (27.2) | 3024 (40.8) | 40.455 | ＜0.001 |
| QDC, n (%) | 445 (5.6) | 44 (7.8) | 401 (5.4) | 5.754 | 0.016 |
| YaDC, n (%) | 1131 (14.2) | 93 (16.5) | 1038 (14.0) | 2.727 | 0.099 |
| YiDC, n (%) | 1000 (12.5) | 97 (17.2) | 903 (12.2) | 12.168 | ＜0.001 |
| PDC, n (%) | 1336 (16.7) | 117 (20.8) | 1219 (16.4) | 7.068 | 0.008 |
| DHC, n (%) | 114 (1.4) | 8 (1.4) | 106 (1.4) | 0.000 | 0.987 |
| BSC, n (%) | 442 (5.5) | 26 (4.6) | 416 (5.6) | 0.986 | 0.321 |
| QSC, n (%) | 270 (3.4) | 19 (3.4) | 251 (3.4) | 0.000 | 0.989 |
| ISC, n (%) | 62 (0.8) | 6 (1.1) | 56 (0.8) | 0.654 | 0.419 |
| χ^2^ | 50.223 | | | | |
| *P*-value | ＜0.001 | | | | |

BC, balanced constitution; QDC, qi-deficiency constitution; YaDC, yang-deficiency constitution; YiDC, yin-deficiency constitution; PDC, phlegm-dampness constitution; DHC, dampness-heat constitution; BSC, blood stasis constitution; QSC, qi stagnation constitution; ISC, inherited special constitution.

**Supplementary Table S5** Distribution of TCM constitutions in older adults aged ≥80 years with and without CMM.

| TCM constitution | Total  (n=2732) | CMM | | χ^2^ | *P*-value |
| --- | --- | --- | --- | --- | --- |
|  |  | Yes (n=186) | No (n=2546) |  |  |
| BC, n (%) | 984 (36.0) | 48 (25.8) | 936 (36.8) | 9.030 | 0.003 |
| QDC, n (%) | 270 (9.9) | 22 (11.8) | 248 (9.7) | 0.848 | 0.357 |
| YaDC, n (%) | 414 (15.2) | 44 (23.7) | 370 (14.5) | 11.221 | 0.001 |
| YiDC, n (%) | 377 (13.8) | 33 (17.7) | 344 (13.5) | 2.608 | 0.106 |
| PDC, n (%) | 311 (11.4) | 24 (12.9) | 287 (11.3) | 0.457 | 0.499 |
| DHC, n (%) | 33 (1.2) | 2 (1.1) | 31 (1.2) | 0.029 | 0.864 |
| BSC, n (%) | 195 (7.1) | 7 (3.8) | 188 (7.4) | 3.428 | 0.064 |
| QSC, n (%) | 115 (4.2) | 6 (3.2) | 109 (4.3) | 0.479 | 0.489 |
| ISC, n (%) | 33 (1.2) | 0 (0.0) | 33 (1.3) | 1.475 | 0.225 |
| χ^2^ | 24.798 | | | | |
| *P*-value | 0.002 | | | | |

BC, balanced constitution; QDC, qi-deficiency constitution; YaDC, yang-deficiency constitution; YiDC, yin-deficiency constitution; PDC, phlegm-dampness constitution; DHC, dampness-heat constitution; BSC, blood stasis constitution; QSC, qi stagnation constitution; ISC, inherited special constitution.

**Supplementary Table S6** Balanced covariates before and after the IPTW using the propensity score for whether there was the QDC.

| Characteristics | Before IPTW | | | | After IPTW | | | |
| --- | --- | --- | --- | --- | --- | --- | --- | --- |
|  | Non-QDC | QDC | *P* | SMD | Non-QDC | QDC | *P* | SMD |
| n | 23614 | 1198 |  |  | 24816 | 24814 |  |  |
| Gender, n (%) |  |  | 0.91 | 0.004 |  |  | 0.472 | 0.027 |
| Female | 13256 (56.1) | 670 (55.9) |  |  | 13930 (56.1) | 13901 (57.5) |  |  |
| Male | 10358 (43.9) | 528 (44.1) |  |  | 10886 (43.9) | 10283 (42.5) |  |  |
| Age, n (%) |  |  | <0.001 | 0.406 |  |  | 0.491 | 0.038 |
| 60-69 | 13620 (57.7) | 483 (40.3) |  |  | 14104 (56.8) | 13927 (57.6) |  |  |
| 70-79 | 7532 (31.9) | 445 (37.1) |  |  | 7976 (32.1) | 7398 (30.6) |  |  |
| ≥80 | 2462 (10.4) | 270 (22.5) |  |  | 2736 (11.0) | 2859 (11.8) |  |  |
| Education, n (%) |  |  | <0.001 | 0.22 |  |  | 0.258 | 0.063 |
| Primary education | 9574 (40.5) | 615 (51.3) |  |  | 10195 (41.1) | 10639 (44.0) |  |  |
| Secondary education | 11380 (48.2) | 462 (38.6) |  |  | 11839 (47.7) | 10805 (44.7) |  |  |
| Tertiary education | 2660 (11.3) | 121 (10.1) |  |  | 2782 (11.2) | 2740 (11.3) |  |  |
| Occupation, n (%) |  |  | <0.001 | 0.244 |  |  | 0.77 | 0.059 |
| Service personnel | 1516 (6.4) | 68 (5.7) |  |  | 1583 (6.4) | 1340 (5.5) |  |  |
| Workers | 9056 (38.4) | 360 (30.1) |  |  | 9414 (37.9) | 8747 (36.2) |  |  |
| Farmers | 7220 (30.6) | 497 (41.5) |  |  | 7722 (31.1) | 7961 (32.9) |  |  |
| Employees and cadres in enterprises and institutions | 2890 (12.2) | 124 (10.4) |  |  | 3015 (12.1) | 3019 (12.5) |  |  |
| Professional and technical personnel | 2269 (9.6) | 108 (9.0) |  |  | 2378 (9.6) | 2352 (9.7) |  |  |
| Other occupations | 663 (2.8) | 41 (3.4) |  |  | 704 (2.8) | 765 (3.2) |  |  |
| Marital status, n (%) |  |  | ＜0.001 | 0.261 |  |  | 0.707 | 0.012 |
| Married | 20537 (87.0) | 923 (77.0) |  |  | 21460 (86.5) | 20817 (86.1) |  |  |
| Unmarried | 3077 (13.0) | 275 (23.0) |  |  | 3356 (13.5) | 3367 (13.9) |  |  |
| Smoking, n (%) |  |  | 0.284 | 0.034 |  |  | 0.323 | 0.039 |
| Yes | 3224 (13.7) | 150 (12.5) |  |  | 3373 (13.6) | 2973 (12.3) |  |  |
| No | 20390 (86.3) | 1048 (87.5) |  |  | 21443 (86.4) | 21211 (87.7) |  |  |
| Drinking, n (%) |  |  | ＜0.001 | 0.134 |  |  | 0.684 | 0.018 |
| Yes | 2745 (11.6) | 92 (7.7) |  |  | 2836 (11.4) | 2627 (10.9) |  |  |
| No | 20869 (88.4) | 1106 (92.3) |  |  | 21980 (88.6) | 21557 (89.1) |  |  |
| Bland taste, n (%) |  |  | 0.01 | 0.076 |  |  | 0.597 | 0.02 |
| Yes | 17764 (75.2) | 861 (71.9) |  |  | 18629 (75.1) | 17945 (74.2) |  |  |
| No | 5850 (24.8) | 337 (28.1) |  |  | 6187 (24.9) | 6239 (25.8) |  |  |
| Spicy taste, n (%) |  |  | 0.689 | 0.013 |  |  | 0.777 | 0.011 |
| Yes | 2855 (12.1) | 150 (12.5) |  |  | 3005 (12.1) | 3016 (12.5) |  |  |
| No | 20759 (87.9) | 1048 (87.5) |  |  | 21811 (87.9) | 21168 (87.5) |  |  |
| Sweet taste, n (%) |  |  | ＜0.001 | 0.178 |  |  | 0.704 | 0.012 |
| Yes | 2601 (11.0) | 206 (17.2) |  |  | 2809 (11.3) | 2829 (11.7) |  |  |
| No | 21013 (89.0) | 992 (82.8) |  |  | 22007 (88.7) | 21355 (88.3) |  |  |
| Salty taste, n (%) |  |  | 0.323 | 0.03 |  |  | 0.633 | 0.018 |
| Yes | 3317 (14.0) | 181 (15.1) |  |  | 3499 (14.1) | 3564 (14.7) |  |  |
| No | 20297 (86.0) | 1017 (84.9) |  |  | 21317 (85.9) | 20620 (85.3) |  |  |
| RPA, n (%) |  |  | ＜0.001 | 0.18 |  |  | 0.723 | 0.015 |
| Yes | 4963 (21.0) | 170 (14.2) |  |  | 5133 (20.7) | 5149 (21.3) |  |  |
| No | 18651 (79.0) | 1028 (85.8) |  |  | 19683 (79.3) | 19035 (78.7) |  |  |
| Sleep quality, n (%) |  |  | ＜0.001 | 0.529 |  |  | 0.202 | 0.047 |
| Good | 13370 (56.6) | 374 (31.2) |  |  | 13743 (55.4) | 12825 (53.0) |  |  |
| Poor | 10244 (43.4) | 824 (68.8) |  |  | 11073 (44.6) | 11359 (47.0) |  |  |
| Depression, n (%) |  |  | ＜0.001 | 0.65 |  |  | 0.157 | 0.039 |
| Yes | 4288 (18.2) | 565 (47.2) |  |  | 4859 (19.6) | 5110 (21.1) |  |  |
| No | 19326 (81.8) | 633 (52.8) |  |  | 19957 (80.4) | 19074 (78.9) |  |  |
| Anxiety, n (%) |  |  | ＜0.001 | 0.454 |  |  | 0.31 | 0.027 |
| Yes | 2834 (12.0) | 360 (30.1) |  |  | 3199 (12.9) | 3341 (13.8) |  |  |
| No | 20780 (88.0) | 838 (69.9) |  |  | 21617 (87.1) | 20843 (86.2) |  |  |
| Hyperlipidemia, n (%) |  |  | 0.299 | 0.033 |  |  | 0.714 | 0.014 |
| Yes | 1529 (6.5) | 68 (5.7) |  |  | 1598 (6.4) | 1477 (6.1) |  |  |
| No | 22085 (93.5) | 1130 (94.3) |  |  | 23218 (93.6) | 22707 (93.9) |  |  |
| Hypertension, n (%) |  |  | 0.001 | 0.101 |  |  | 0.882 | 0.005 |
| Yes | 8266 (35.0) | 478 (39.9) |  |  | 8746 (35.2) | 8460 (35.0) |  |  |
| No | 15348 (65.0) | 720 (60.1) |  |  | 16070 (64.8) | 15724 (65.0) |  |  |
| CKD, n (%) |  |  | 0.001 | 0.086 |  |  | 0.837 | 0.006 |
| Yes | 228 (1.0) | 24 (2.0) |  |  | 251 (1.0) | 232 (1.0) |  |  |
| No | 23386 (99.0) | 1174 (98.0) |  |  | 24565 (99.0) | 23952 (99.0) |  |  |

RPA, regular physical activity; CKD, chronic kidney disease.

**Supplementary Table S7** Balanced covariates before and after the IPTW using the propensity score for whether there was the YaDC.

| Characteristics | Before IPTW | | | | After IPTW | | | |
| --- | --- | --- | --- | --- | --- | --- | --- | --- |
|  | Non- YaDC | YaDC | *P* | SMD | Non- YaDC | YaDC | *P* | SMD |
| n | 21455 | 3357 |  |  | 24814 | 24778 |  |  |
| Gender, n (%) |  |  | ＜0.001 | 0.253 |  |  | 0.596 | 0.011 |
| Female | 11686 (54.5) | 2240 (66.7) |  |  | 13931 (56.1) | 14048(56.7) |  |  |
| Male | 9769 (45.5) | 1117 (33.3) |  |  | 10883 (43.9) | 10730 (43.3) |  |  |
| Age, n (%) |  |  | 0.001 | 0.07 |  |  | 0.319 | 0.031 |
| 60-69 | 12291 (57.3) | 1812 (54.0) |  |  | 14095 (56.8) | 13703 (55.3) |  |  |
| 70-79 | 6846 (31.9) | 1131 (33.7) |  |  | 7984 (32.2) | 8216 (33.2) |  |  |
| ≥80 | 2318 (10.8) | 414 (12.3) |  |  | 2735 (11.0) | 2859 (11.5) |  |  |
| Education, n (%) |  |  | 0.003 | 0.062 |  |  | 0.952 | 0.006 |
| Primary education | 8800 (41.0) | 1389 (41.4) |  |  | 10191 (41.1) | 10252 (41.4) |  |  |
| Secondary education | 10303 (48.0) | 1539 (45.8) |  |  | 11842 (47.7) | 11767 (47.5) |  |  |
| Tertiary education | 2352 (11.0) | 429 (12.8) |  |  | 2781 (11.2) | 2759 (11.1) |  |  |
| Occupation, n (%) |  |  | ＜0.001 | 0.095 |  |  | 0.977 | 0.018 |
| Service personnel | 1364 (6.4) | 220 (6.6) |  |  | 1583 (6.4) | 1552 (6.3) |  |  |
| Workers | 8139 (37.9) | 1277 (38.0) |  |  | 9413 (37.9) | 9307 (37.6) |  |  |
| Farmers | 6744 (31.4) | 973 (29.0) |  |  | 7721 (31.1) | 7794 (31.5) |  |  |
| Employees and cadres in enterprises and institutions | 2626 (12.2) | 388 (11.6) |  |  | 3011 (12.1) | 2936 (11.8) |  |  |
| Professional and technical personnel | 2004 (9.3) | 373 (11.1) |  |  | 2381 (9.6) | 2453 (9.9) |  |  |
| Other occupations | 578 (2.7) | 126 (3.8) |  |  | 705 (2.8) | 736 (3.0) |  |  |
| Marital status, n (%) |  |  | ＜0.001 | 0.101 |  |  | 0.608 | 0.01 |
| Married | 18661 (87.0) | 2799 (83.4) |  |  | 21459 (86.5) | 21345 (86.1) |  |  |
| Unmarried | 2794 (13.0) | 558 (16.6) |  |  | 3355 (13.5) | 3433 (13.9) |  |  |
| Smoking, n (%) |  |  | ＜0.001 | 0.077 |  |  | 0.85 | 0.004 |
| Yes | 2992 (13.9) | 382 (11.4) |  |  | 3373 (13.6) | 3333 (13.5) |  |  |
| No | 18463 (86.1) | 2975 (88.6) |  |  | 21441 (86.4) | 21445 (86.5) |  |  |
| Drinking, n (%) |  |  | ＜0.001 | 0.113 |  |  | 0.96 | 0.001 |
| Yes | 2552 (11.9) | 285 (8.5) |  |  | 2837 (11.4) | 2842 (11.5) |  |  |
| No | 18903 (88.1) | 3072 (91.5) |  |  | 21977 (88.6) | 21936 (88.5) |  |  |
| Bland taste, n (%) |  |  | 0.354 | 0.018 |  |  | 0.394 | 0.018 |
| Yes | 16083 (75.0) | 2542 (75.7) |  |  | 18622 (75.0) | 18403 (74.3) |  |  |
| No | 5372 (25.0) | 815 (24.3) |  |  | 6192 (25.0) | 6375 (25.7) |  |  |
| Spicy taste, n (%) |  |  | 0.611 | 0.01 |  |  | 0.303 | 0.022 |
| Yes | 2589 (12.1) | 416 (12.4) |  |  | 3012 (12.1) | 3184 (12.9) |  |  |
| No | 18866 (87.9) | 2941 (87.6) |  |  | 21802 (87.9) | 21594 (87.1) |  |  |
| Sweet taste, n (%) |  |  | 0.001 | 0.059 |  |  | 0.689 | 0.008 |
| Yes | 2372 (11.1) | 435 (13.0) |  |  | 2813 (11.3) | 2871 (11.6) |  |  |
| No | 19083 (88.9) | 2922 (87.0) |  |  | 22001 (88.7) | 21907 (88.4) |  |  |
| Salty taste, n (%) |  |  | 0.006 | 0.05 |  |  | 0.516 | 0.013 |
| Yes | 2973 (13.9) | 525 (15.6) |  |  | 3504 (14.1) | 3612 (14.6) |  |  |
| No | 18482 (86.1) | 2832 (84.4) |  |  | 21310 (85.9) | 21166 (85.4) |  |  |
| RPA, n (%) |  |  | ＜0.001 | 0.082 |  |  | 0.631 | 0.009 |
| Yes | 4340 (20.2) | 793 (23.6) |  |  | 5130 (20.7) | 5028 (20.3) |  |  |
| No | 17115 (79.8) | 2564 (76.4) |  |  | 19684 (79.3) | 19750 (79.7) |  |  |
| Sleep quality, n (%) |  |  | ＜0.001 | 0.454 |  |  | 0.876 | 0.003 |
| Good | 12526 (58.4) | 1218 (36.3) |  |  | 13743 (55.4) | 13685 (55.2) |  |  |
| Poor | 8929 (41.6) | 2139 (63.7) |  |  | 11071 (44.6) | 11093 (44.8) |  |  |
| Depression, n (%) |  |  | ＜0.001 | 0.246 |  |  | 0.934 | 0.001 |
| Yes | 3897 (18.2) | 956 (28.5) |  |  | 4853 (19.6) | 4832 (19.5) |  |  |
| No | 17558 (81.8) | 2401 (71.5) |  |  | 19961 (80.4) | 19946 (80.5) |  |  |
| Anxiety, n (%) |  |  | ＜0.001 | 0.092 |  |  | 0.062 | 0.036 |
| Yes | 2669 (12.4) | 525 (15.6) |  |  | 3203 (12.9) | 3507 (14.2) |  |  |
| No | 18786 (87.6) | 2832 (84.4) |  |  | 21611 (87.1) | 21271 (85.8) |  |  |
| Hyperlipidemia, n (%) |  |  | 0.27 | 0.021 |  |  | 0.623 | 0.011 |
| Yes | 1396 (6.5) | 201 (6.0) |  |  | 1599 (6.4) | 1661 (6.7) |  |  |
| No | 20059 (93.5) | 3156 (94.0) |  |  | 23215 (93.6) | 23117 (93.3) |  |  |
| Hypertension, n (%) |  |  | 0.157 | 0.027 |  |  | 0.93 | 0.002 |
| Yes | 7524 (35.1) | 1220 (36.3) |  |  | 8747 (35.3) | 8756 (35.3) |  |  |
| No | 13931 (64.9) | 2137 (63.7) |  |  | 16067 (64.7) | 16022 (64.7) |  |  |
| CKD, n (%) |  |  | 0.035 | 0.038 |  |  | 0.991 | ＜0.001 |
| Yes | 206 (1.0) | 46 (1.4) |  |  | 252 (1.0) | 251 (1.0) |  |  |
| No | 21249 (99.0) | 3311 (98.6) |  |  | 24562 (99.0) | 24527 (99.0) |  |  |

RPA, regular physical activity; CKD, chronic kidney disease.

**Supplementary Table S8** Balanced covariates before and after the IPTW using the propensity score for whether there was the YiDC.

| Characteristics | Before IPTW | | | | After IPTW | | | |
| --- | --- | --- | --- | --- | --- | --- | --- | --- |
|  | Non- YiDC | YiDC | *P* | SMD | Non- YiDC | YiDC | *P* | SMD |
| n | 21939 | 2873 |  |  | 24817 | 24593 |  |  |
| Gender, n (%) |  |  | 0.011 | 0.051 |  |  | 0.901 | 0.003 |
| Female | 12249 (55.8) | 1677 (58.4) |  |  | 13928 (56.1) | 13834 (56.3) |  |  |
| Male | 9690 (44.2) | 1196 (41.6) |  |  | 10889 (43.9) | 10759 (43.7) |  |  |
| Age, n (%) |  |  | ＜0.001 | 0.113 |  |  | 0.816 | 0.013 |
| 60-69 | 12607 (57.5) | 1496 (52.1) |  |  | 14101 (56.8) | 13903 (56.5) |  |  |
| 70-79 | 6977 (31.8) | 1000 (34.8) |  |  | 7980 (32.2) | 7879 (32.0) |  |  |
| ≥80 | 2355 (10.7) | 377 (13.1) |  |  | 2736 (11.0) | 2811 (11.4) |  |  |
| Education, n (%) |  |  | 0.003 | 0.067 |  |  | 0.909 | 0.009 |
| Primary education | 8926 (40.7) | 1263 (44.0) |  |  | 10190 (41.1) | 10020 (40.7) |  |  |
| Secondary education | 10545 (48.1) | 1297 (45.1) |  |  | 11844 (47.7) | 11750 (47.8) |  |  |
| Tertiary education | 2468 (11.2) | 313 (10.9) |  |  | 2783 (11.2) | 2823 (11.5) |  |  |
| Occupation, n (%) |  |  | ＜0.001 | 0.124 |  |  | 0.986 | 0.017 |
| Service personnel | 1409 (6.4) | 175 (6.1) |  |  | 1585 (6.4) | 1580 (6.4) |  |  |
| Workers | 8423 (38.4) | 993 (34.6) |  |  | 9415 (37.9) | 9131 (37.1) |  |  |
| Farmers | 6679 (30.4) | 1038 (36.1) |  |  | 7720 (31.1) | 7771 (31.6) |  |  |
| Employees and cadres in enterprises and institutions | 2694 (12.3) | 320 (11.1) |  |  | 3015 (12.1) | 3032 (12.3) |  |  |
| Professional and technical personnel | 2106 (9.6) | 271 (9.4) |  |  | 2378 (9.6) | 2380 (9.7) |  |  |
| Other occupations | 628 (2.9) | 76 (2.6) |  |  | 704 (2.8) | 699 (2.8) |  |  |
| Marital status, n (%) |  |  | ＜0.001 | 0.102 |  |  | 0.615 | 0.01 |
| Married | 19067 (86.9) | 2393 (83.3) |  |  | 21461 (86.5) | 21184 (86.1) |  |  |
| Unmarried | 2872 (13.1) | 480 (16.7) |  |  | 3356 (13.5) | 3409 (13.9) |  |  |
| Smoking, n (%) |  |  | 0.004 | 0.057 |  |  | 0.338 | 0.02 |
| Yes | 2933 (13.4) | 441 (15.3) |  |  | 3379 (13.6) | 3515 (14.3) |  |  |
| No | 19006 (86.6) | 2432 (84.7) |  |  | 21438 (86.4) | 21078 (85.7) |  |  |
| Drinking, n (%) |  |  | 0.575 | 0.012 |  |  | 0.389 | 0.018 |
| Yes | 2499 (11.4) | 338 (11.8) |  |  | 2841 (11.5) | 2959 (12.0) |  |  |
| No | 19440 (88.6) | 2535 (88.2) |  |  | 21976 (88.5) | 21634 (88.0) |  |  |
| Bland taste, n (%) |  |  | ＜0.001 | 0.117 |  |  | 0.292 | 0.021 |
| Yes | 16600 (75.7) | 2025 (70.5) |  |  | 18622 (75.0) | 18227 (74.1) |  |  |
| No | 5339 (24.3) | 848 (29.5) |  |  | 6195 (25.0) | 6366 (25.9) |  |  |
| Spicy taste, n (%) |  |  | ＜0.001 | 0.126 |  |  | 0.559 | 0.011 |
| Yes | 2547 (11.6) | 458 (15.9) |  |  | 3008 (12.1) | 3071 (12.5) |  |  |
| No | 19392 (88.4) | 2415 (84.1) |  |  | 21809 (87.9) | 21522 (87.5) |  |  |
| Sweet taste, n (%) |  |  | ＜0.001 | 0.083 |  |  | 0.374 | 0.018 |
| Yes | 2413 (11.0) | 394 (13.7) |  |  | 2812 (11.3) | 2925 (11.9) |  |  |
| No | 19526 (89.0) | 2479 (86.3) |  |  | 22005 (88.7) | 21668 (88.1) |  |  |
| Salty taste, n (%) |  |  | ＜0.001 | 0.078 |  |  | 0.287 | 0.021 |
| Yes | 3022 (13.8) | 476 (16.6) |  |  | 3505 (14.1) | 3658 (14.9) |  |  |
| No | 18917 (86.2) | 2397 (83.4) |  |  | 21312 (85.9) | 20935 (85.1) |  |  |
| RPA, n (%) |  |  | 0.008 | 0.052 |  |  | 0.302 | 0.021 |
| Yes | 4484 (20.4) | 649 (22.6) |  |  | 5139 (20.7) | 5305 (21.6) |  |  |
| No | 17455 (79.6) | 2224 (77.4) |  |  | 19678 (79.3) | 19288 (78.4) |  |  |
| Sleep quality, n (%) |  |  | ＜0.001 | 0.331 |  |  | 0.424 | 0.016 |
| Good | 12567 (57.3) | 1177 (41.0) |  |  | 13741 (55.4) | 13415 (54.5) |  |  |
| Poor | 9372 (42.7) | 1696 (59.0) |  |  | 11076 (44.6) | 11178 (45.5) |  |  |
| Depression, n (%) |  |  | ＜0.001 | 0.297 |  |  | 0.283 | 0.02 |
| Yes | 3971 (18.1) | 882 (30.7) |  |  | 4861 (19.6) | 5009 (20.4) |  |  |
| No | 17968 (81.9) | 1991 (69.3) |  |  | 19956 (80.4) | 19584 (79.6) |  |  |
| Anxiety, n (%) |  |  | ＜0.001 | 0.175 |  |  | 0.149 | 0.027 |
| Yes | 2665 (12.1) | 529 (18.4) |  |  | 3201 (12.9) | 3399 (13.8) |  |  |
| No | 19274 (87.9) | 2344 (81.6) |  |  | 21616 (87.1) | 21194 (86.2) |  |  |
| Hyperlipidemia, n (%) |  |  | 0.208 | 0.025 |  |  | 0.885 | 0.003 |
| Yes | 1396 (6.4) | 201 (7.0) |  |  | 1597 (6.4) | 1565 (6.4) |  |  |
| No | 20543 (93.6) | 2672 (93.0) |  |  | 23220 (93.6) | 23028 (93.6) |  |  |
| Hypertension, n (%) |  |  | ＜0.001 | 0.137 |  |  | 0.57 | 0.012 |
| Yes | 7563 (34.5) | 1181 (41.1) |  |  | 8750 (35.3) | 8807 (35.8) |  |  |
| No | 14376 (65.5) | 1692 (58.9) |  |  | 16067 (64.7) | 15786 (64.2) |  |  |
| CKD, n (%) |  |  | ＜0.001 | 0.069 |  |  | 0.807 | 0.004 |
| Yes | 203 (0.9) | 49 (1.7) |  |  | 253 (1.0) | 261 (1.1) |  |  |
| No | 21736 (99.1) | 2824 (98.3) |  |  | 24564 (99.0) | 24332 (98.9) |  |  |

RPA, regular physical activity; CKD, chronic kidney disease.

**Supplementary Table S9** Balanced covariates before and after the IPTW using the propensity score for whether there was the PDC.

| Characteristics | Before IPTW | | | | After IPTW | | | |
| --- | --- | --- | --- | --- | --- | --- | --- | --- |
|  | Non- PDC | PDC | *P* | SMD | Non- PDC | PDC | *P* | SMD |
| n | 20927 | 3885 |  |  | 24813 | 24823 |  |  |
| Gender, n (%) |  |  | ＜0.001 | 0.214 |  |  | 0.77 | 0.005 |
| Female | 12093 (57.8) | 1833 (47.2) |  |  | 13921 (56.1) | 13859 (55.8) |  |  |
| Male | 8834 (42.2) | 2052 (52.8) |  |  | 10892 (43.9) | 10964 (44.2) |  |  |
| Age, n (%) |  |  | ＜0.001 | 0.124 |  |  | 0.803 | 0.013 |
| 60-69 | 11865 (56.7) | 2238 (57.6) |  |  | 14095 (56.8) | 14007 (56.4) |  |  |
| 70-79 | 6641 (31.7) | 1336 (34.4) |  |  | 7984 (32.2) | 7980 (32.1) |  |  |
| ≥80 | 2421 (11.6) | 311 (8.0) |  |  | 2734 (11.0) | 2836(11.4) |  |  |
| Education, n (%) |  |  | 0.228 | 0.03 |  |  | 0.913 | 0.008 |
| Primary education | 8619 (41.2) | 1570 (40.4) |  |  | 10199 (41.1) | 10303 (41.5) |  |  |
| Secondary education | 9942 (47.5) | 1900 (48.9) |  |  | 11834 (47.7) | 1176 (47.4) |  |  |
| Tertiary education | 2366 (11.3) | 415 (10.7) |  |  | 2780 (11.2) | 2760 (11.1) |  |  |
| Occupation, n (%) |  |  | ＜0.001 | 0.081 |  |  | 0.56 | 0.037 |
| Service personnel | 1283 (6.1) | 301 (7.7) |  |  | 1585 (6.4) | 1608 (6.5) |  |  |
| Workers | 7917 (37.8) | 1499 (38.6) |  |  | 9405 (37.9) | 9106 (36.7) |  |  |
| Farmers | 6558 (31.3) | 1159 (29.8) |  |  | 7733 (31.2) | 8074 (32.5) |  |  |
| Employees and cadres in enterprises and institutions | 2548 (12.2) | 466 (12.0) |  |  | 3002 (12.1) | 2860 (11.5) |  |  |
| Professional and technical personnel | 2041 (9.8) | 336 (8.6) |  |  | 2383 (9.6) | 2448 (9.9) |  |  |
| Other occupations | 580 (2.8) | 124 (3.2) |  |  | 705 (2.8) | 727 (2.9) |  |  |
| Marital status, n (%) |  |  | 0.275 | 0.02 |  |  | 0.885 | 0.003 |
| Married | 18078 (86.4) | 3382 (87.1) |  |  | 21459 (86.5) | 21443 (86.4) |  |  |
| Unmarried | 2849 (13.6) | 503 (12.9) |  |  | 3354 (13.5) | 3379 (13.6) |  |  |
| Smoking, n (%) |  |  | ＜0.001 | 0.31 |  |  | 0.587 | 0.009 |
| Yes | 2464 (11.8) | 910 (23.4) |  |  | 3385 (13.6) | 3460 (13.9) |  |  |
| No | 18463 (88.2) | 2975 (76.6) |  |  | 21428 (86.4) | 21363 (86.1) |  |  |
| Drinking, n (%) |  |  | ＜0.001 | 0.32 |  |  | 0.933 | 0.001 |
| Yes | 2019 (9.6) | 818 (21.1) |  |  | 2840 (11.4) | 2831 (11.4) |  |  |
| No | 18908 (90.4) | 3067 (78.9) |  |  | 21973 (88.6) | 21992 (88.6) |  |  |
| Bland taste, n (%) |  |  | ＜0.001 | 0.198 |  |  | 0.342 | 0.017 |
| Yes | 15999 (76.5) | 2626 (67.6) |  |  | 18610 (75.0) | 18435 (74.3) |  |  |
| No | 4928 (23.5) | 1259 (32.4) |  |  | 6203 (25.0) | 6388 (25.7) |  |  |
| Spicy taste, n (%) |  |  | ＜0.001 | 0.124 |  |  | 0.422 | 0.014 |
| Yes | 2396 (11.4) | 609 (15.7) |  |  | 3014 (12.1) | 3130 (12.6) |  |  |
| No | 18531 (88.6) | 3276 (84.3) |  |  | 21799 (87.9) | 21693 (87.4) |  |  |
| Sweet taste, n (%) |  |  | ＜0.001 | 0.115 |  |  | 0.685 | 0.007 |
| Yes | 2243 (10.7) | 564 (14.5) |  |  | 2810 (11.3) | 2867 (11.6) |  |  |
| No | 18684 (89.3) | 3321 (85.5) |  |  | 22003 (88.7) | 21956 (88.4) |  |  |
| Salty taste, n (%) |  |  | ＜0.001 | 0.145 |  |  | 0.703 | 0.007 |
| Yes | 2777 (13.3) | 721 (18.6) |  |  | 3507 (14.1) | 3566 (14.4) |  |  |
| No | 18150 (86.7) | 3164 (81.4) |  |  | 21306 (85.9) | 21257 (85.6) |  |  |
| RPA, n (%) |  |  | ＜0.001 | 0.101 |  |  | 0.437 | 0.014 |
| Yes | 4192 (20.0) | 941 (24.2) |  |  | 5123 (20.6) | 4986 (20.1) |  |  |
| No | 16735 (80.0) | 2944 (75.8) |  |  | 19690 (79.4) | 19837 (79.9) |  |  |
| Sleep quality, n (%) |  |  | ＜0.001 | 0.102 |  |  | 0.511 | 0.013 |
| Good | 11427 (54.6) | 2317 (59.6) |  |  | 13735 (55.4) | 13586 (54.7) |  |  |
| Poor | 9500 (45.4) | 1568 (40.4) |  |  | 11078 (44.6) | 11237 (45.3) |  |  |
| Depression, n (%) |  |  | ＜0.001 | 0.083 |  |  | 0.354 | 0.019 |
| Yes | 4199 (20.1) | 654 (16.8) |  |  | 4857 (19.6) | 5044 (20.3) |  |  |
| No | 16728 (79.9) | 3231 (83.2) |  |  | 19956 (80.4) | 19779 (79.7) |  |  |
| Anxiety, n (%) |  |  | 0.81 | 0.005 |  |  | 0.516 | 0.013 |
| Yes | 2699 (12.9) | 495 (12.7) |  |  | 3196 (12.9) | 3303 (13.3) |  |  |
| No | 18228 (87.1) | 3390 (87.3) |  |  | 21617 (87.1) | 21520 (86.7) |  |  |
| Hyperlipidemia, n (%) |  |  | ＜0.001 | 0.208 |  |  | 0.726 | 0.005 |
| Yes | 1159 (5.5) | 438 (11.3) |  |  | 1594 (6.4) | 1563 (6.3) |  |  |
| No | 19768 (94.5) | 3447 (88.7) |  |  | 23219 (93.6) | 23260 (93.7) |  |  |
| Hypertension, n (%) |  |  | ＜0.001 | 0.282 |  |  | 0.982 | ＜0.001 |
| Yes | 6927 (33.1) | 1817 (46.8) |  |  | 8745 (35.2) | 8744 (35.2) |  |  |
| No | 14000 (66.9) | 2068 (53.2) |  |  | 16068 (64.8) | 16079 (64.8) |  |  |
| CKD, n (%) |  |  | 0.056 | 0.037 |  |  | 0.908 | 0.002 |
| Yes | 224 (1.1) | 28 (0.7) |  |  | 253 (1.0) | 247 (1.0) |  |  |
| No | 20703 (98.9) | 3857 (99.3) |  |  | 24560 (99.0) | 24576 (99.0) |  |  |

RPA, regular physical activity; CKD, chronic kidney disease.
